# Heme Oxygenase-1 and Its Metabolites Carbon Monoxide and Biliverdin, but Not Iron, Exert Antiviral Activity against Porcine Circovirus Type 3

**DOI:** 10.1128/spectrum.05060-22

**Published:** 2023-05-04

**Authors:** Lei Hou, Xiaoyu Yang, Changzhe Liu, Jinshuo Guo, Yongyan Shi, Tong Sun, Xufei Feng, Jianwei Zhou, Jue Liu

**Affiliations:** a College of Veterinary Medicine, Yangzhou University, Yangzhou, China; b Jiangsu Co-Innovation Center for Prevention and Control of Important Animal Infectious Diseases and Zoonoses, Yangzhou University, Yangzhou, China; University of Prince Edward Island

**Keywords:** porcine circovirus type 3, heme oxygenase-1, carbon monoxide, biliverdin, viral replication

## Abstract

Porcine circovirus type 3 (PCV3) is a newly discovered pathogen that causes porcine dermatitis and nephropathy syndrome (PDNS)-like clinical signs, multisystemic inflammation, and reproductive failure. Heme oxygenase-1 (HO-1), a stress-inducible enzyme, exerts protective functions by converting heme into carbon monoxide (CO), biliverdin (BV), and iron. However, the effects of HO-1 and its metabolites on PCV3 replication remain unknown. In this study, experiments involving specific inhibitors, lentivirus transduction, and small interfering RNA (siRNA) transfection revealed that active PCV3 infection reduced HO-1 expression and that the expression of HO-1 negatively regulated virus replication in cultured cells, depending on its enzymatic activity. Subsequently, the effects of the HO-1 metabolites (CO, BV, and iron) on PCV3 infection were investigated. The CO inducers (cobalt protoporphyrin IX [CoPP] or tricarbonyl dichloro ruthenium [II] dimer [CORM-2]) mediate PCV3 inhibition by generating CO, and this inhibition is reversed by hemoglobin (Hb; a CO scavenger). The inhibition of PCV3 replication by BV depended on BV-mediated reactive oxygen species (ROS) reduction, as *N*-acetyl-l-cysteine affected PCV3 replication while reducing ROS production. The reduction product of BV, bilirubin (BR), specifically promoted nitric oxide (NO) generation and further activated the cyclic GMP/protein kinase G (cGMP/PKG) pathway to attenuate PCV3 infection. Both the iron provided by FeCl_3_ and the iron chelated by deferoxamine (DFO) with CoPP treatment failed to affect PCV3 replication. Our data demonstrate that the HO-1-CO-cGMP/PKG, HO-1-BV-ROS, and HO-1-BV-BR-NO-cGMP/PKG pathways contribute crucially to the inhibition of PCV3 replication. These results provide important insights regarding preventing and controlling PCV3 infection.

**IMPORTANCE** The regulation of host protein expression by virus infection is the key to facilitating self-replication. As an important emerging pathogen of swine, clarification of the interaction between PCV3 infection and the host enables us to understand the viral life cycle and pathogenesis better. Heme oxygenase-1 (HO-1) and its metabolites carbon monoxide (CO), biliverdin (BV), and iron have been demonstrated to involve a wealth of viral replications. Here, we, for the first time, demonstrated that HO-1 expression decreases in PCV3-infected cells and negatively regulates PCV3 replication and that the HO-1 metabolic products CO and BV inhibit PCV3 replication by the CO- or BV/BR/NO-dependent cGMP/PKG pathway or BV-mediated ROS reduction, but the iron (the third metabolic product) does not. Specifically, PCV3 infection maintains normal proliferation by downregulating HO-1 expression. These findings clarify the mechanism by which HO-1 modulates PCV3 replication in cells and provide important targets for preventing and controlling PCV3 infection.

## INTRODUCTION

Porcine circovirus type 3 (PCV3) is a novel porcine circovirus that was first identified in sows with porcine dermatitis and nephropathy syndrome (PDNS)-like clinical signs, multisystemic inflammation, and reproductive failure in the United States in 2015 (1, 2), and it was subsequently identified in many other countries around the world (3–10) and has resulted in substantial economic losses worldwide. Retrospective research in China has demonstrated that PCV3 infection can be traced back nearly half a century (11, 12). In 2019, PDNS-like disease was observed in piglets infected with PCV3 generated by DNA infectious clones (13), and the gut microbiota was dynamically altered by PCV3 infection (14), confirming that PCV3 is pathogenic to pigs and contributes to the initiation of PDNS-like clinical signs. *In vitro* studies have reported the mechanisms underlying the interaction between PCV3 infection and the host (15). For example, PCV3 enters PK-15 cells via a pH-dependent, clathrin- and dynamin-2-mediated endocytosis that requires Rab5 and Rab7 (16). Nucleolar phosphoprotein nucleophosmin-1 (NPM1) facilitates PCV3 replication by interacting with viral cap proteins (17). Additionally, the regulatory function of the PCV3 cap protein in host cells was partially clarified (18–20).

The heme oxygenase (HMOX or HO) gene family encodes three isoforms of HO-1, HO-2, and HO-3. HO-1 is primarily expressed in the liver, spleen, bone marrow, and gastrointestinal tract. The rapid increase in HO-1 protects against oxidative stress, inflammation, or other adverse stimuli (21–23). HO-2 is expressed in the testes, brain, liver, and gut (21, 24, 25). HO-3 is a pseudogene with enzymatic activity (26).

HO-1, a stress-inducible enzyme, has attracted attention as an important endogenous protective factor to eliminate various cellular stresses such as oxidative stress, endotoxins, hypoxia, heavy metals, heat shock, and inflammatory cytokines (27–29). Certain studies have reported that HO-1 performs protective functions due to its ability to degrade heme. In this process, the by-products of heme degradation (CO, iron, and BV and its reduction product BR) mediate its cytoprotective effects in response to oxidative dysregulation, aberrant immune response, and related disorders (30). First, CO is involved in attenuating inflammation and regulating cell proliferation, apoptosis, and other cellular processes (31). Second, BR is derived from BV (another metabolite of HO-1) through biliverdin reductase (BVR) and acted as a potent antioxidant to clear reactive oxygen species (ROS), thus suppressing lipid and protein peroxidation (32). BR also plays an important role in controlling inflammation and adaptive immunoreactions (33). Third, free iron induces several reactive radicals by reacting with hydrogen or lipid peroxides, ultimately increasing the risk of tissue injury and disease (34). As HO-1 is utilized by cells to neutralize stress responses, the induction of this stress-inducible enzyme is regarded as an effective therapeutic strategy to resist various diseases. Viral infection often stimulates oxidative stress. Recent research data have also shown that HO-1 plays a crucial role in antiviral activity (35). For example, the upregulation of HO-1 inhibited hepatitis B virus (HBV) (36), human respiratory syncytial virus (RSV) (37), human immunodeficiency virus (HIV) (38), reproductive and respiratory syndrome virus (PRRSV) (39), and dengue virus (DENV) (40). However, the effect of HO-1 on PCV3 replication and its regulatory mechanisms remains unknown.

In this study, we first explored the relationship between PCV3 infection and HO-1 expression and observed that PCV3 infection decreased HO-1 expression and that changes in HO-1 expression negatively regulated PCV3 replication. The effects of HO-1 metabolic products (CO, BV and its reduction product BR, and free iron) on PCV3 replication were further investigated. The results revealed that CO significantly inhibited PCV3 replication by activating the cyclic GMP (cGMP)/protein kinase G (PKG) pathway, and BV and BR attenuated PCV3 infection through ROS reduction and the NO-dependent cGMP/PKG pathway; however, iron, the third HO-1 metabolite, did not exhibit an inhibitory role in PCV3 replication. These findings indicate that HO-1 and its metabolites are involved in PCV3 replication in PK-15 cells and provide important targets for preventing and controlling PCV3 infection.

## RESULTS

### PCV3 infection reduces the expression of HO-1 in PK-15 cells.

**FIG 1:**
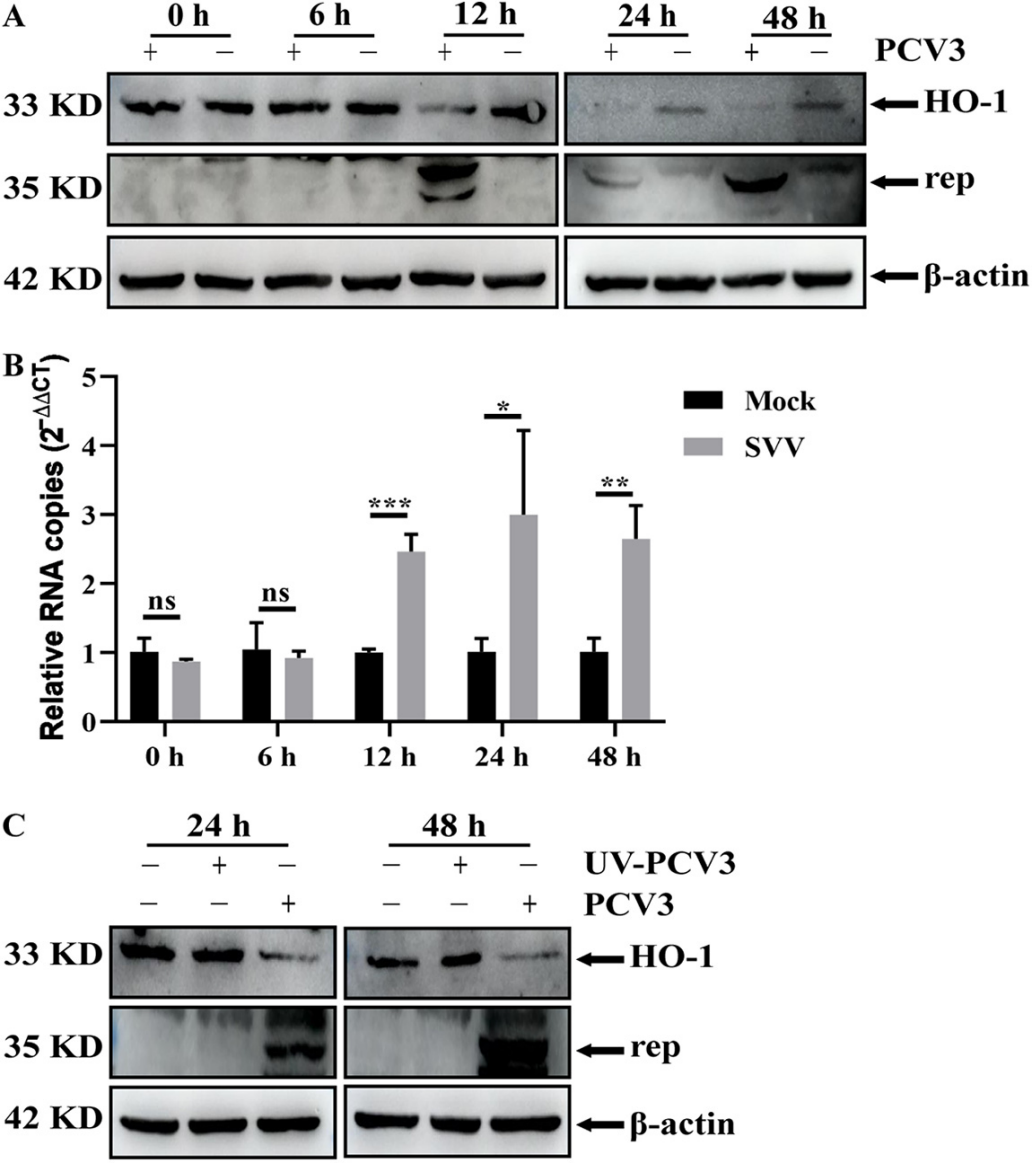
PCV3 infection reduces HO-1 expression in PK-15 cells. (A and B) Proteins and HO-1 mRNA were extracted from PCV3-infected or mock-infected PK-15 cells at 0, 6, 12, 24, and 48 hpi and were analyzed by Western blotting with anti-HO-1 and anti-Rep antibodies (A) and RT-qPCR (B), respectively. β-Actin or GAPDH was used as a control. (C) PK-15 cells were infected with PCV3 or UV-inactivated PCV3 (MOI of 0.5) for 24 or 48 h. Subsequently, proteins were extracted and analyzed as described in panel A. Data are presented as the means ± standard deviations (SD) from three independent experiments (ns, *P* > 0.05; *, *P* < 0.05; **, *P* < 0.01; ***, *P* < 0.001).

HO-1 plays a crucial role in cellular protection. To explore the relationship between PCV3 infection and HO-1, we first measured if the expression and transcription levels of HO-1 were affected by PCV3 infection in PK-15 cells at various time points using Western blotting and reverse transcriptase quantitative PCR (RT-qPCR). The results revealed that PCV3 infection significantly decreased the expression level of HO-1 protein and increased the transcription level of mRNA compared to the mock-infected group, and this change was time dependent (Fig. 1A and B). UV-inactivated viruses are considered to lose infectivity in cells. Thus, we used UV-inactivated PCV3-infected cells to explore if the decrease in HO-1 expression was dependent on active PCV3 replication. As presented in Fig. 1C, UV-inactivated PCV3 did not change HO-1 expression compared to that in PCV3-infected cells at 24 or 48 h postinfection (hpi), and this was similar to the mock-infected group, indicating that active PCV3 replication is required for the decrease in HO-1 expression.

### Upregulation of HO-1 expression induced by CoPP specifically inhibits PCV3 replication.

**FIG 2:**
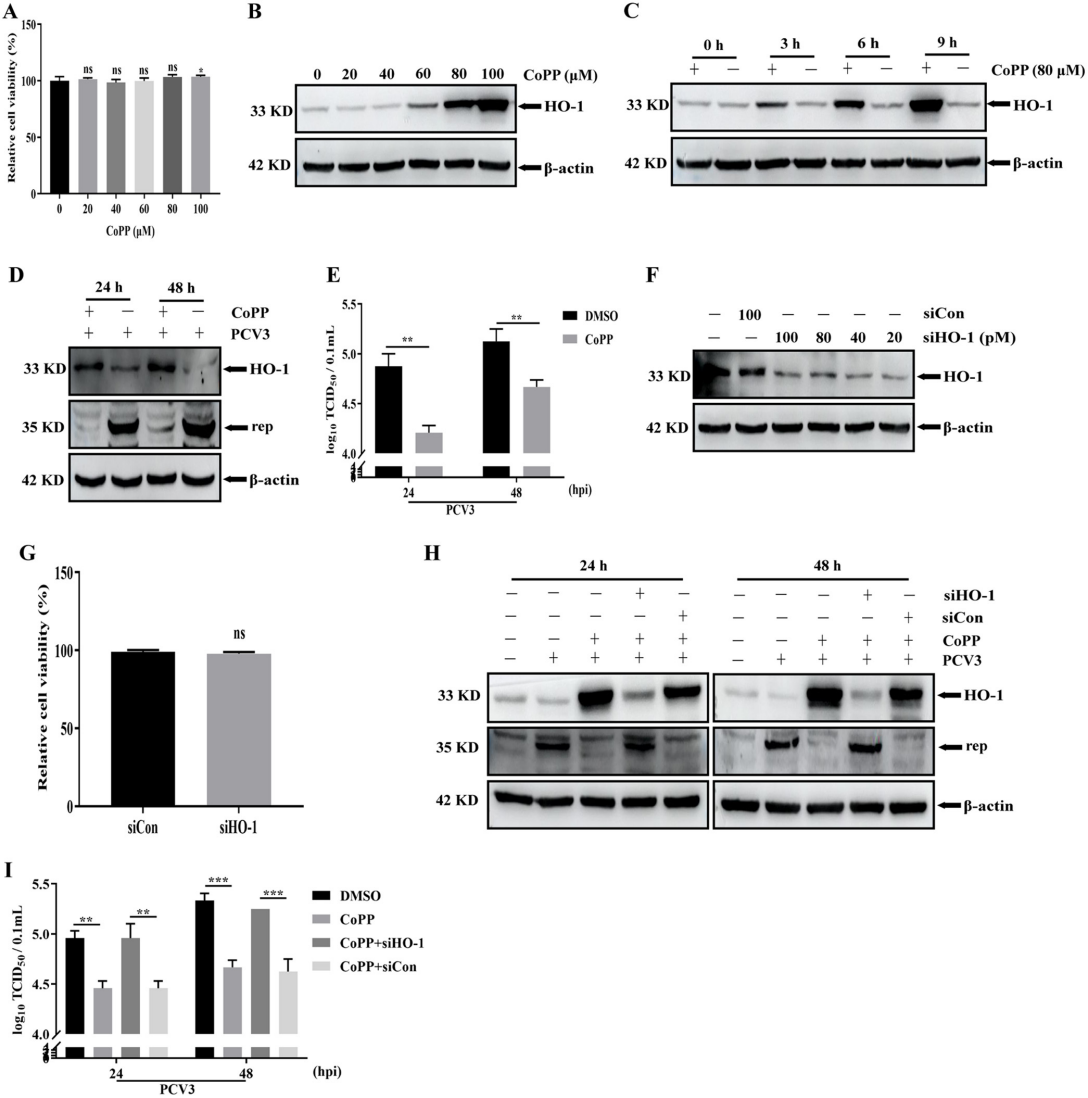
Upregulation of HO-1 expression induced by CoPP treatment inhibits PCV3 replication. (A) Cell viability after exposure to different concentrations of CoPP (20, 40, 60, 80, and 100 μM) or DMSO (represented by 0 μM CoPP) was evaluated using CCK8 assays. (B) PK-15 cells were incubated with various concentrations of CoPP or DMSO for 24 h, and proteins were extracted and analyzed. (C) PK-15 cells were incubated with CoPP (80 μM) or DMSO for different lengths of time, and proteins were extracted and analyzed. (D and E) PK-15 cells were pretreated with CoPP (80 μM) for 6 h and then subjected to PCV3 (MOI of 0.5) infection, and cell culture media were replaced with media containing or lacking CoPP. After 24 or 48 hpi, the cells and whole-cell culture media were collected and then measured for Rep protein (D) and PCV3 titers (E). (F) PK-15 cells were transfected with siHO-1 at various concentrations (20, 40, 80, and 100 pM) or with 100 pM siCon. HO-1 proteins were detected by Western blotting at 48 h. (G) Cell viability between siHO-1 (40 pM) and siCon (40 pM) was evaluated USING CCK8 assays. (H and I) PK-15 cells transfected with siHO-1 or siCon were pretreated with CoPP for 6 h and then subjected to PCV3 infection, and cell culture media were replaced with media containing or lacking CoPP. After 24 or 48 hpi, the cells and whole-cell culture media were collected and then measured for Rep protein (H) and PCV3 titers (I). Data are presented as the means ± SD from three independent experiments (ns, *P* > 0.05; *, *P* < 0.05; **, *P* < 0.01; ***, *P* < 0.001).

Cobalt protoporphyrin IX (CoPP) is a specific inducer of HO-1 expression of HO-1 in host cells (28, 39). Cell viability was first detected using Cell Counting Kit-8 (CCK8) assays in CoPP-treated PK-15 cells to analyze the cytotoxicity of the inducer. Cell viability was not affected at concentrations of up to 80 μM CoPP at 24 h (Fig. 2A). Subsequently, the expression of HO-1 was analyzed in PK-15 cells treated with different concentrations of CoPP. Western blotting results revealed that HO-1 expression exhibited an upward trend with increasing CoPP concentration (Fig. 2B). Moreover, the appropriate time for CoPP treatment was screened. Sufficient expression of HO-1 was observed after CoPP treatment for 6 h compared to the control group. This time point was finally selected for subsequent experiments (Fig. 2C). PCV3 infection reduced HO-1 expression, prompting us to explore the effect of the change in HO-1 expression on the PCV3 replication. PK-15 cells pretreated with CoPP for 6 h were infected with PCV3, and CoPP was present during PCV3 infection. As presented in Fig. 2D, CoPP treatment notably increased HO-1 expression and reduced the expression of the viral Rep protein at 24 and 48 hpi. Additionally, the decrease in PCV3 titers by CoPP treatment confirmed the above-described results (Fig. 2E), indicating that CoPP treatment inhibited PCV3 replication. To determine whether HO-1 specifically mediated the inhibitory activity of CoPP, PK-15 cells were transfected with various concentrations of small interfering RNA (siRNA) targeting the HO-1 gene (siHO-1), and the silencing effect of HO-1 was then analyzed using Western blotting. The results revealed that HO-1 expression was decreased by siHO-1 transfection (from 20 pM to 100 pM) compared to levels in the control group (siCon) (Fig. 2F). The cell viability was not affected at concentrations of 40 pM siHO-1(Fig. 2G), and the 40 pM siHO-1 with a notable silencing effect was finally selected for subsequent experiments. Next, PK-15 cells transfected with siHO-1 or siCon were infected with PCV3 and treated with CoPP, and the expression of HO-1, viral Rep protein, and viral titers was analyzed by Western blotting or viral titer assay. The results demonstrated that siHO-1 reversed the CoPP-mediated reduction of the Rep protein (Fig. 2H), and the viral titer results also confirmed this reversal role of siHO-1 compared to the control group (Fig. 2I), indicating that HO-1 specifically induces the antiviral role of CoPP.

### HO-1 expression negatively regulates PCV3 replication.

**FIG 3:**
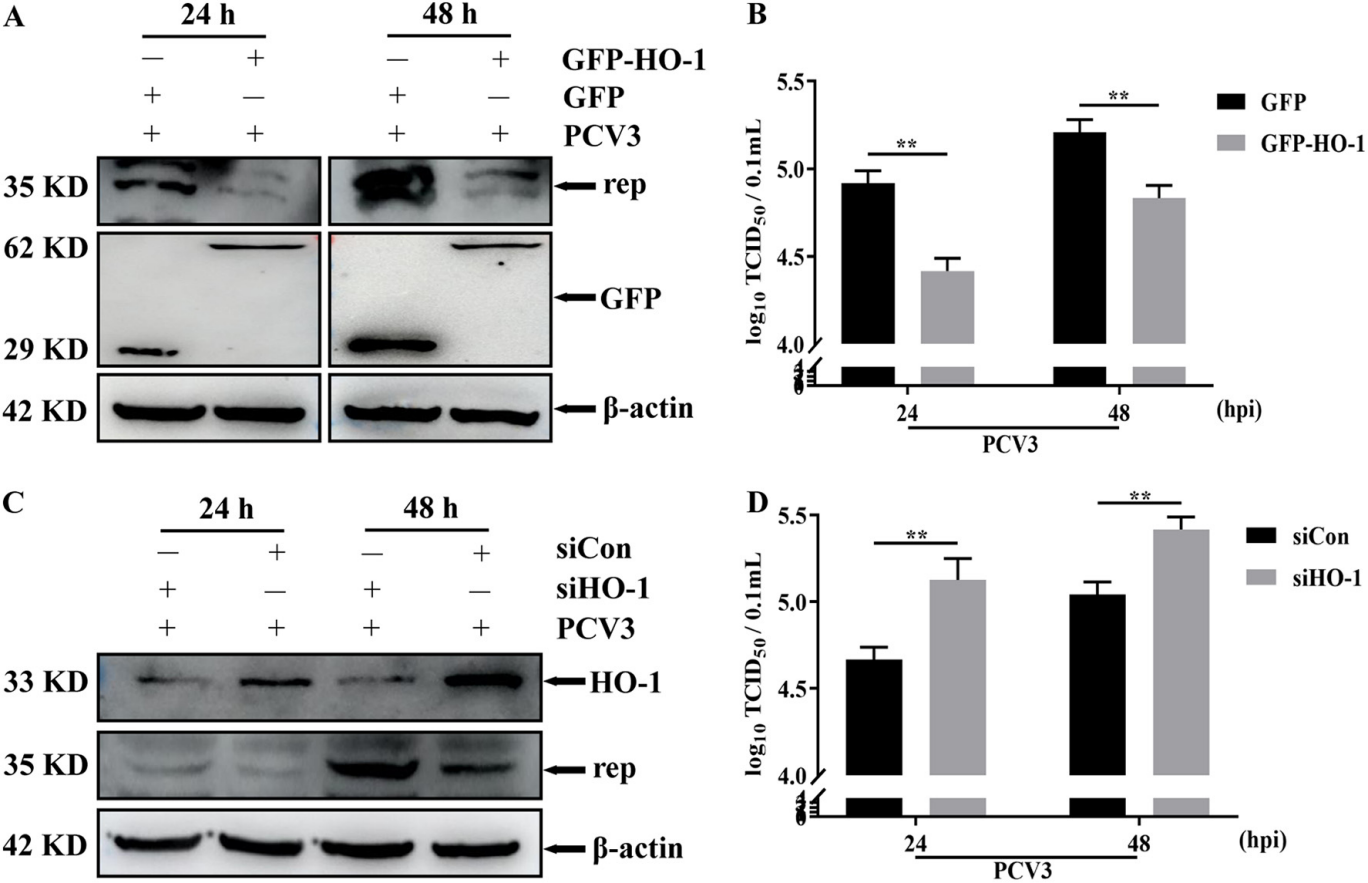
The effect of the change of HO-1 expression on PCV3 replication. (A and B) PK-15 cell lines stably overexpressing GFP-HO-1 or GFP infected with PCV3; cells and whole-cell culture media were collected at 24 and 48 hpi and measured for Rep protein (A) and PCV3 titers (B). (C and D) PK-15 cells were transfected with siHO-1 (40 pM) or siCon (40 pM) for 48 h and then subjected to PCV3 infection, and cells and whole-cell culture media were subsequently collected at 24 and 48 hpi and measured for Rep protein (C) and PCV3 titers (D). Data are presented as the means ± SD from three independent experiments (**, *P* < 0.01).

To further confirm the effect of CoPP-dependent HO-1 on viral replication, we extended the above-described studies by exploring the effect of HO-1 expression (HO-1 overexpression or knockdown) on PCV3 replication. The PK-15 cell line stably overexpressing green fluorescent protein (GFP)-HO-1 or GFP was constructed using a recombinant lentivirus delivery system. As presented in Fig. S1A in the supplemental material, PK-15 cells were successfully infected and transduced with lentivirus expressing GFP-HO-1 or GFP, and single-cloned cells expressing GFP-HO-1 and GFP were screened, cultured, and validated by indirect fluorescent-antibody assay (IFA). Western blotting results further demonstrated that GFP-HO-1 protein or GFP protein was overexpressed in the cloned cells (Fig. S1B), and no obvious differences in cell viability were observed between GFP-HO-1-expressing and GFP-expressing PK-15 cells (Fig. S1C). Based on the above-described construction of the HO-1 overexpression cell line, the effect of HO-1 overexpression on PCV3 replication at different time points (24 and 48 hpi) was further evaluated via Western blotting and a viral titer assay. HO-1 overexpression significantly reduced the expression of viral Rep protein and viral titers compared to those in the control group (Fig. 3A and B). Subsequently, the effect of HO-1 knockdown by siRNA targeting HO-1 on PCV3 replication was further analyzed. As presented in Fig. 3C and D, the silencing of HO-1 by siHO-1 significantly increased the expression of Rep protein and PCV3 titers. These results suggested that HO-1 expression negatively regulates PCV3 replication.

### The enzyme activity of HO-1 plays a crucial role in antiviral function.

**FIG 4:**
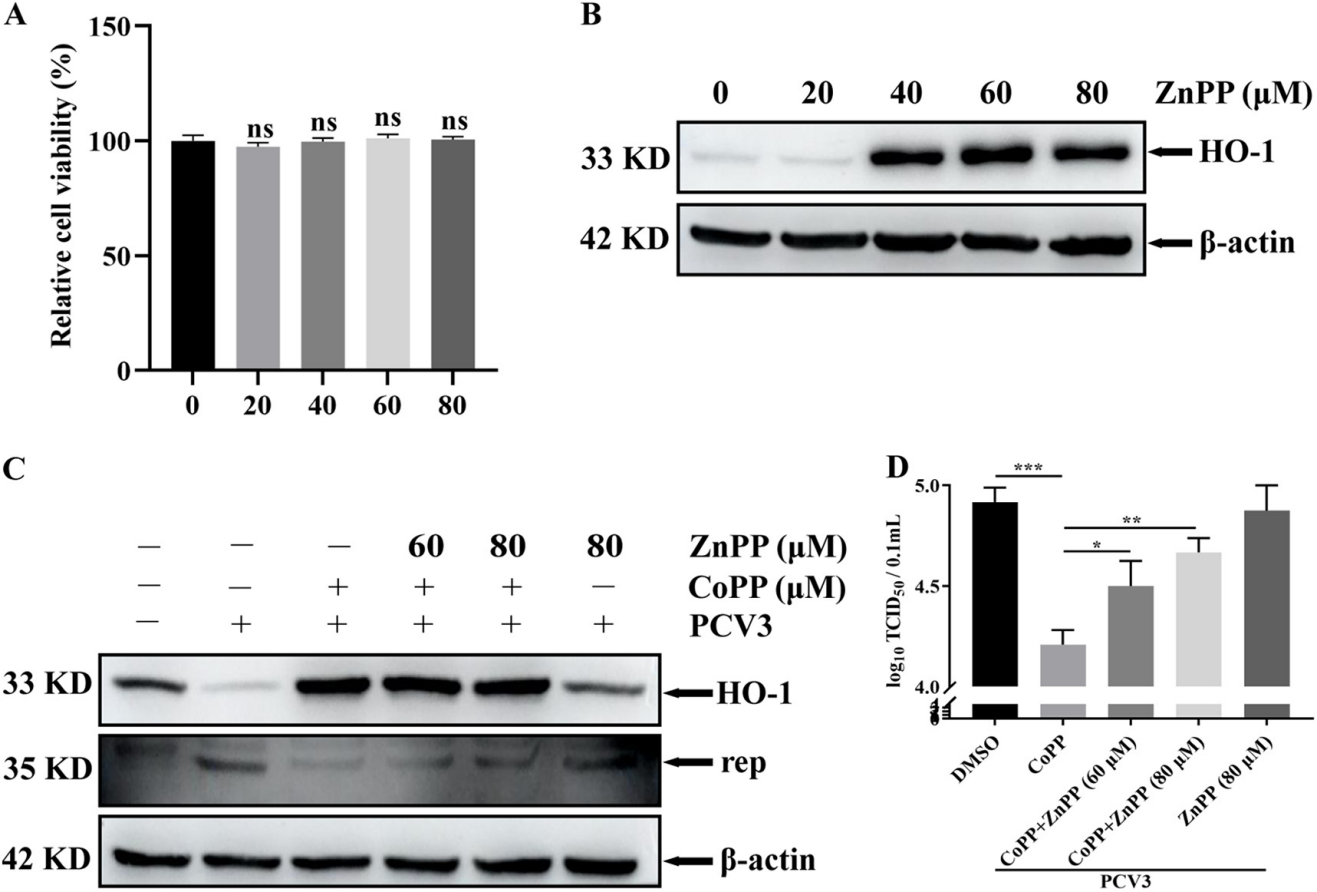
The effect of enzyme activity of HO-1 on PCV3 replication. (A) Cell viability after exposure to various concentrations of ZnPP (20, 40, 60, and 80 μM) or DMSO (represented by 0 μM ZnPP) was evaluated using CCK8 assays. (B) PK-15 was treated with 0, 20, 40, 60, and 80 μM ZnPP for 24 h. Subsequently, proteins were extracted and analyzed as described in Fig. 1A. (C and D) PK-15 was pretreated with ZnPP (60 or 80 μM) or CoPP (80 μM) and then subjected to PCV3 infection, and cell culture media were subsequently replaced with media containing or lacking ZnPP or CoPP. After 24 hpi, the cells and whole-cell culture media were collected and then measured for Rep protein (C) and PCV3 titers (D). Data are presented as the means ± SD from three independent experiments (ns, *P* > 0.05; *, *P* < 0.05; **, *P* < 0.01; ***, *P* < 0.001).

HO-1, a metabolic enzyme ubiquitously distributed in mammalian cells, can catalyze the conversion of heme to CO, BV, and free iron (41). To explore if the antiviral role of HO-1 is dependent on its enzymatic activity, zinc protoporphyrin (ZnPP), an inhibitor of HO-1 enzyme activity, was used to evaluate if the inhibitory role of HO-1 in PCV3 replication was related to its enzymatic activity. The effect of ZnPP on cell viability was first analyzed, and the results revealed that ZnPP at concentrations of up to 80 μM did not affect cell viability (Fig. 4A). Subsequently, the expression of HO-1 was analyzed in PK-15 cells treated with various concentrations of ZnPP (0, 20, 40, 60, and 80 μM) for 24 h. The results revealed that ZnPP treatment starting from 40 μM increased the expression of HO-1 (Fig. 4B), and 60 or 80 μM ZnPP was used in subsequent experiments. PK-15 cells infected with PCV3 were treated with CoPP and ZnPP for 24 h. The results demonstrated that ZnPP treatment increased the expression of viral Rep protein (Fig. 4C) and viral titers (Fig. 4D) in a dose-dependent fashion even when HO-1 expression was elevated, and this partially reversed the inhibitory role of CoPP-induced HO-1 in PCV3 replication. These results indicate that the enzymatic activity of HO-1 is essential for inhibiting PCV3 replication.

### CO, an HO-1 metabolite, mediates the anti-PCV3 function of HO-1.

**FIG 5:**
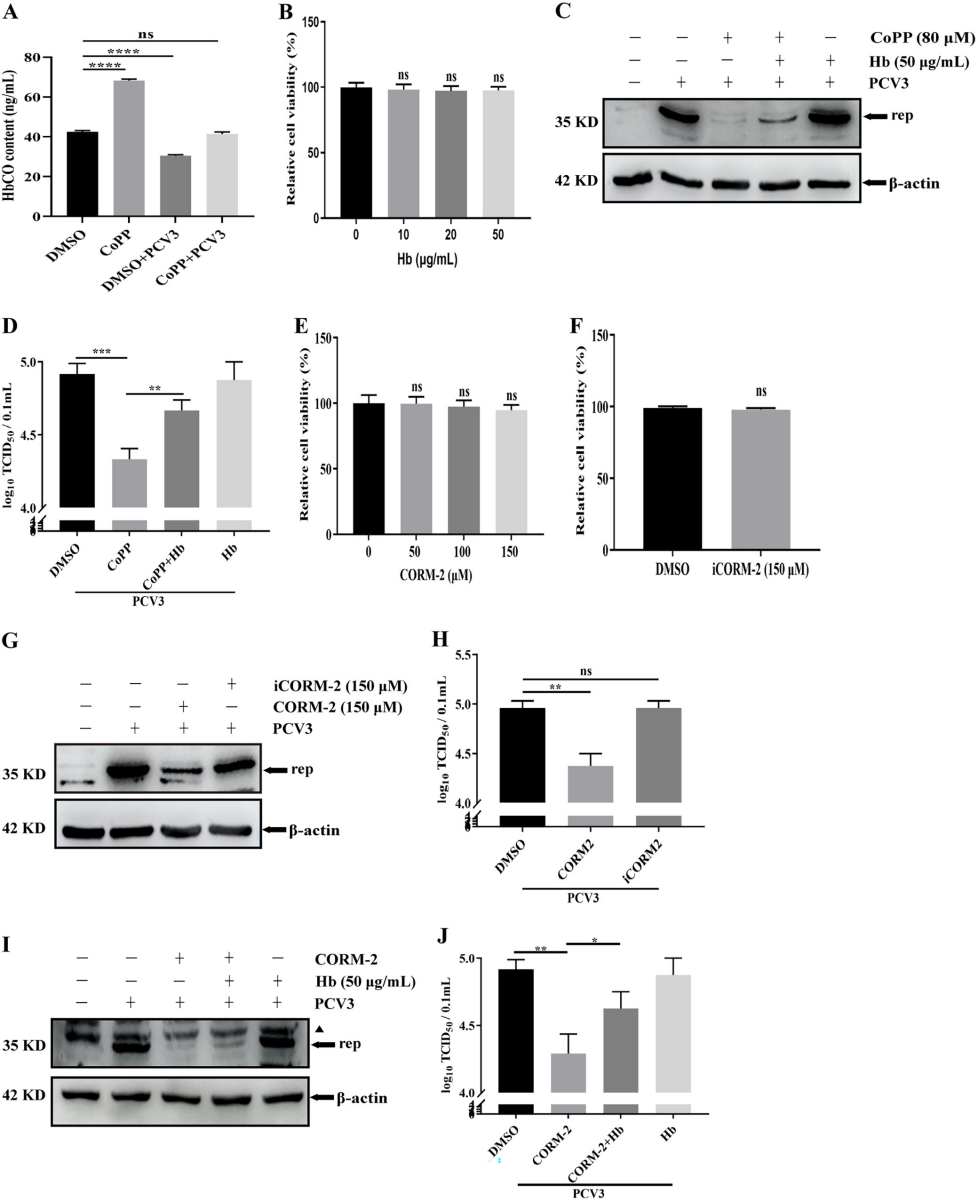
The inhibitory effect of CO on PCV3 replication. (A) PK-15 cells treated or untreated with CoPP were infected or uninfected with PCV3 for 24 h, and then cell culture supernatants were collected to quantify HbCO contents by ELISA as a measure of CO. (B) Cell viability after exposure to various concentrations of Hb (10, 20, and 50 μg/mL) or water (represented by 0 μg/mL of Hb) was evaluated using CCK8 assays. (C and D) PK-15 cells were treated or untreated with the Hb (50 μg/mL) or CoPP and then subjected to PCV3 infection, and the cells and whole-cell culture media were subsequently collected at 24 hpi and measured for Rep protein (C) and PCV3 titers (D). (E and F) Cell viability after exposure to various concentrations of CORM-2 (50, 100, and 150 μM), iCORM-2 (150 μM), or DMSO (represented by 0 μM CORM-2) was evaluated using CCK8 assays. (G and H) PK-15 cells were treated or untreated with the CORM-2 or iCORM-2 and then subjected to PCV3 infection, and the cells and whole-cell culture media were subsequently collected at 24 hpi and measured for Rep protein (G) and PCV3 titers (H). (I and J) PK-15 cells were treated or untreated with the Hb (50 μg/mL) or CORM-2 and then subjected to PCV3 infection, and the cells and whole-cell culture media were subsequently collected at 24 hpi and measured for Rep protein (I) and PCV3 titers (J). The black triangle represents a nonspecific protein band. Data are presented as means ± SD from three independent experiments (ns, *P* > 0.05; *, *P* < 0.05; **, *P* < 0.01; ***, *P* < 0.001; ****, *P* < 0.0001).

Based on the effect of the enzymatic activity of HO-1 on inhibiting PCV3 replication, CO is one of the three downstream metabolites induced by HO-1 (42), and this prompted us to explore the relationship between CO treatment and PCV3 replication. The intracellular CO content was analyzed using the hemoglobin CO (HbCO) assay measure to CO in PCV3-infected or mock-infected PK-15 cells treated or untreated with CoPP for 24 h. Enzyme-linked immunosorbent assay (ELISA) results demonstrated that PCV3 infection reduced intracellular CO content, and CoPP treatment as a positive control of CO production markedly enhanced CO content and partially reversed the PCV3-mediated CO content decrease (Fig. 5A), indicating that CO may be related to CoPP-mediated PCV3 inhibition. To confirm this hypothesis, Hb, a CO scavenger (43, 44), was used to assess the effects of CO on PCV3 replication. PK-15 cells were treated with different concentrations of Hb for 24 h, and no toxicity was observed according to the cell viability assay (Fig. 5B). Subsequently, PK-15 cells were pretreated with Hb (50 μg/mL) for 1 h, and this was followed by PCV3 infection and CoPP treatment in the presence or absence of Hb for 24 h. As presented in Fig. 5C, in the presence of CoPP, the expression of the viral Rep protein increased in PK-15 cells treated with Hb compared to that in Hb-untreated cells. Moreover, the viral titers were consistent with the above-described results, confirming that Hb treatment weakened the inhibitory effect of CoPP on PCV3 replication by removing CO (Fig. 5D). These results suggested that CO, as a metabolite of HO-1, participates in the inhibition of PCV3 replication.

Carbon monoxide-releasing molecule-2 (CORM-2), a specific CO donor, was used as a positive inducer of CO production to confirm the anti-PCV3 function of CO. PK-15 cells treated with various concentrations of CORM-2 (50, 100, and 150 μM) or iCORM-2 (an inactive variant of CORM-2; 150 μM) for 24 h were analyzed using a cell viability assay. The results revealed no differences in cell viability (Fig. 5E and F). Therefore, 150 μM CORM-2 was selected for subsequent experiments. PK-15 cells were pretreated with CORM-2 or iCORM-2 for 1 h, followed by PCV3 infection for 24 h in the presence of CORM-2 or iCORM-2. Western blotting and a viral 50% tissue culture infective dose (TCID_50_) assay were conducted to measure the expression level of viral Rep protein and PCV3 titers. As presented in Fig. 5G and H, CORM-2 treatment markedly reduced rep protein expression and PCV3 titers compared to those in the CORM-2-untreated group, and iCORM-2 treatment did not lead to a significant inhibitory effect on Rep expression and viral titers compared to those of the control group. To further confirm the antiviral effect of CORM-2-mediated CO production on PCV3 replication, Hb treatment was used to evaluate the anti-PCV3 role of CO. As presented in Fig. 5I, Hb treatment alleviated the CORM-2-mediated inhibitory effect on PCV3 replication in PK-15 cells. Moreover, the viral titers were consistent with the above-described results (Fig. 5J). These results indicated that CO specifically exerted the antiviral function of CORM-2 and was used as an HO-1 metabolite to inhibit PCV3 replication.

### The upregulation of HO-1 expression and the activation of the cGMP/PKG pathway mediated by CO involve the inhibitory effect of CO on PCV3 replication.

**FIG 6:**
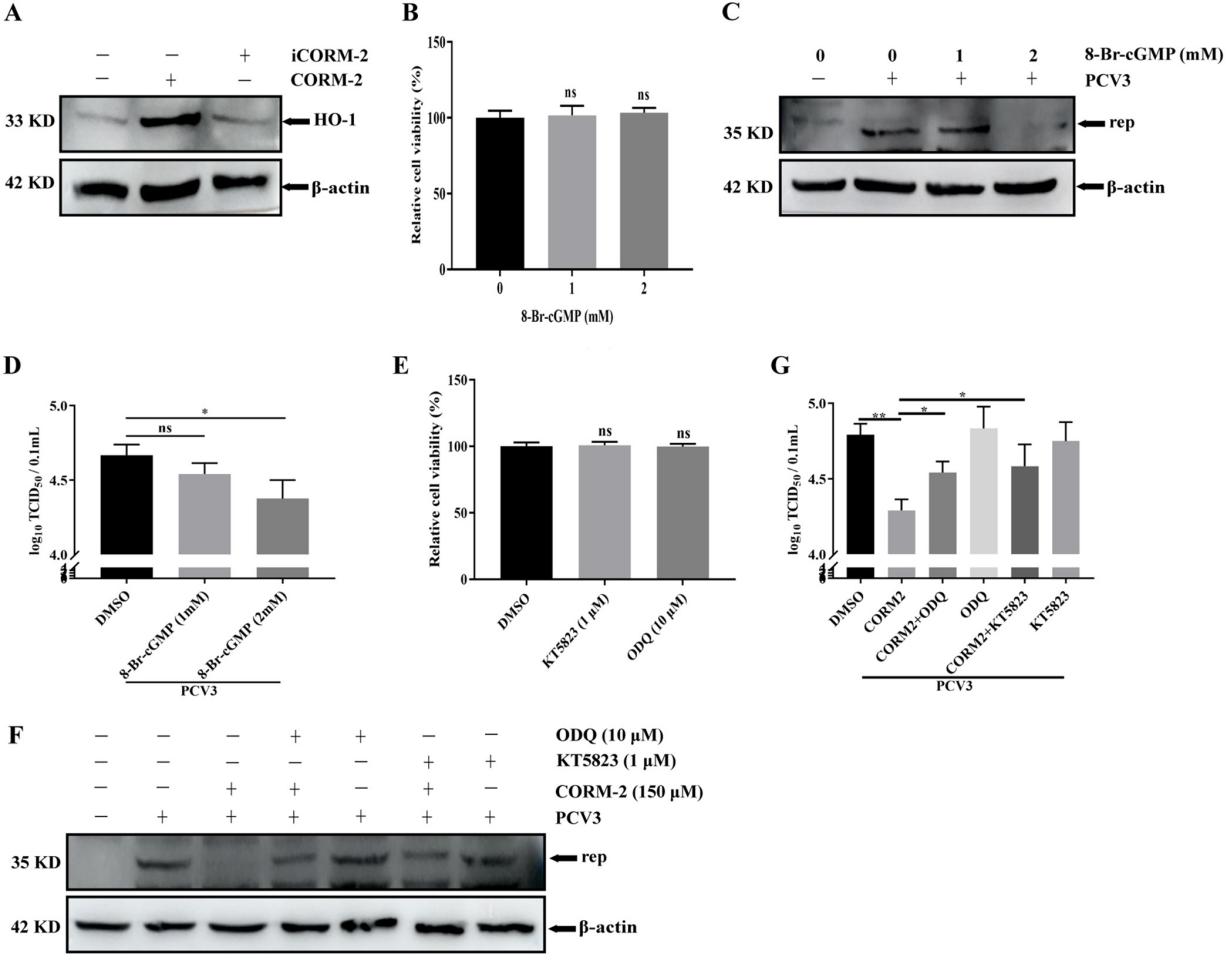
The inhibitory effect of CO on PCV3 replication depends on HO-1 upregulation and the cGMP/PKG pathway. (A) PK-15 cells treated with CORM-2 or iCORM-2 for 24 h were collected and then measured for HO-1 protein. (B) Cell viability after various concentrations of 8-Br-cGMP (1 and 2 mM) or DMSO (represented by 0 mM 8-Br-cGMP) exposure was evaluated using CCK8 assays. (C and D) PK-15 cells were treated or untreated with the 8-Br-cGMP (1 or 2 mM), followed by PCV3 infection, and then the cells and whole-cell culture media were collected at 24 hpi and measured for rep protein (C) and PCV3 titers (D). (E) Cell viability after ODQ (10 μM), KT5823 (1 μM), or DMSO exposure was evaluated using CCK8 assays. (F and G) PK-15 cells were treated or untreated with the ODQ, KT5823, or CORM-2, followed by PCV3 infection, and then the cells and whole-cell culture media were collected at 24 hpi and measured for Rep protein (F) and PCV3 titers (G) (ns, *P* > 0.05; *, *P* < 0.05; **, *P* < 0.01).

The antiviral mechanisms of CO needed to be further investigated. First, we explored the effect of CO on HO-1 expression. Western blotting results demonstrated that CORM-2 treatment increased HO-1 expression and that HO-1 expression in iCORM-2-treated cells did not change compare to that of the mock-treated PK-15 cells (Fig. 6A), indicating that CO induced by CORM-2 has a positive feedback regulation of HO-1 expression, and this regulation is one of the reasons for the antiviral effect of CO. Moreover, a previous study reported that the cGMP/PKG pathway is involved in the CO-mediated antiviral properties (45). Subsequently, the effect of the cGMP/PKG signaling pathway on PCV3 replication was investigated. The cGMP analog 8-Br-cGMP is a positive inducer of the cGMP/PKG pathway, and its treatment has been used to analyze the effect of this signaling pathway on PCV3 replication. Nontoxic concentrations of 8-Br-cGMP in PK-15 cells were screened using a cell viability assay. Adding 8-Br-cGMP up to 2 mM did not affect cell viability (Fig. 6B). Next, PK-15 cells were pretreated with different doses of 8-Br-cGMP (1 or 2 mM) for 24 h. The cells and whole-cell culture medium were collected for further analysis. The results demonstrated that 2 mM 8-Br-cGMP reduced the Rep expression (Fig. 6C) and viral titers (Fig. 6D). We further demonstrated that the inhibition of PCV3 replication by CO depends on the cGMP/PKG pathway. The compounds 1H-[1,2,4] oxadiazole [4,3-α] quinoxaline-1-one (ODQ) and KT5823 are soluble guanylate cyclase (sGC) and protein kinase G (PKG) inhibitors, respectively. Suitable concentrations of these two inhibitors were determined using cell viability assays. The results revealed that 10 μM ODQ and 1 μM KT5823 did not affect cell viability (Fig. 6E), and these concentrations were used in subsequent experiments. PK-15 cells infected with PCV3 were treated with CORM-2, ODQ, or KT5823 to further analyze the effect of these chemical reagents on PCV3 replication. As presented in Fig. 6F and G, treatment with both ODQ and KT5823 partially reversed the CORM-2-mediated inhibition of Rep expression and PCV3 titers, whereas ODQ or KT5823 alone did not exhibit an inhibitory effect on PCV3 replication, indicating that PCV3 replication is dependent on the cGMP/PKG signaling pathway in PK-15 cells. These results suggest that the anti-PCV3 activity of CO is dependent on CO-mediated upregulation of HO-1 and activation of the cGMP/PKG signaling pathway.

### BV, another HO-1 metabolite, and its reduction product BR specifically mediate the anti-PCV3 function of HO-1.

**FIG 7:**
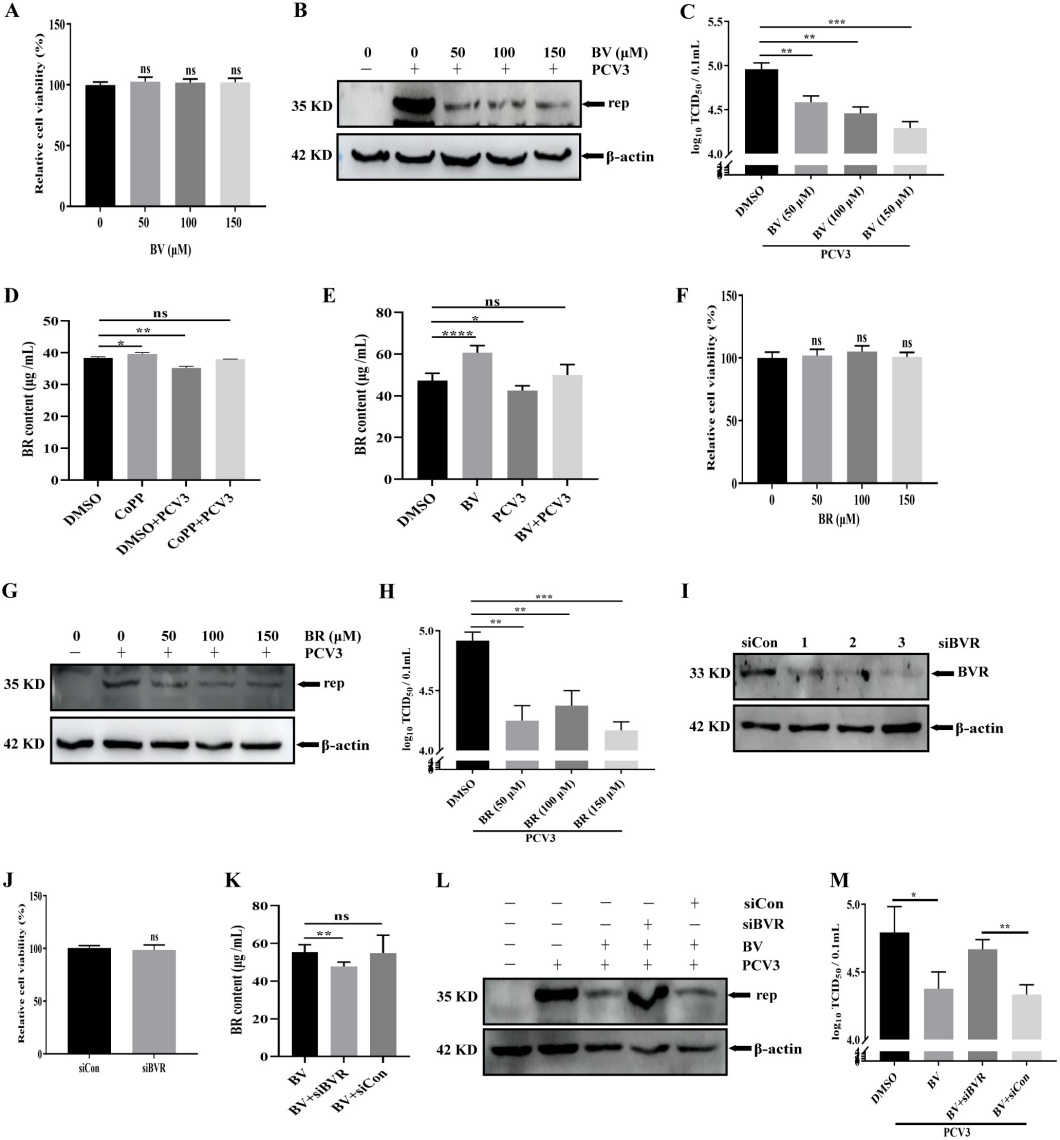
The inhibitory effect of BV and its metabolite, BR, mediated by HO-1 on PCV3 replication. (A) Cell viability after exposure to various concentrations of BV (50, 100, and 150 μM) or DMSO (represented by 0 μM BV) was evaluated using CCK8 assays. (B and C) PK-15 cells were treated or untreated with various concentrations of BV and then subjected to PCV3 infection, and the cells and whole-cell culture media were subsequently collected at 24 hpi and measured for Rep protein (B) and PCV3 titers (C). (D) PK-15 cells treated or untreated with CoPP were infected or uninfected with PCV3 for 24 h, and then cell culture supernatants were harvested to quantify BR contents with ELISA. (E) PK-15 cells treated or untreated with BV were infected or uninfected with PCV3 for 24 h, and then cell culture supernatants were harvested to quantify BR contents with ELISA. (F) Cell viability after exposure to various concentrations of BR (50, 100, and 150 μM) or DMSO (represented by 0 μM BR) was evaluated using CCK8 assays. (G and H) PK-15 cells were treated or untreated with various concentrations of BR and then subjected to PCV3 infection, and the cells and whole-cell culture media were subsequently collected at 24 hpi and measured for Rep protein (G) and PCV3 titers (H). (I) PK-15 cells were transfected with different siBVRs (1, 2, and 3) or siCon for 48 h and then detected by Western blotting with an anti-BVR antibody. (J) Cell viability between siBVR and siCon was evaluated using CCK8 assays. (K) PK-15 cells transfected or untransfected with siBVR or siCon for 48 h were treated with BV. After 24 h, the cell culture supernatants were harvested to quantify BR contents using ELISA. (L and M) PK-15 cells transfected or untransfected with siBVR, or siCon were treated with BV and then subjected to PCV3 infection, and the cells and whole-cell culture media were subsequently collected at 24 hpi and measured for Rep protein (L) and PCV3 titers (M). Data are presented as means ± SD from three independent experiments (ns, *P* > 0.05; *, *P* < 0.05; **, *P* < 0.01; ***, *P* < 0.001; ****, *P* < 0.0001).

As HO-1 activation induces an increase in BV (42) and the enzymatic activity of HO-1 is associated with PCV3 replication, we assessed if BV is involved in the anti-PCV3 function of HO-1. The cytotoxicity of various concentrations of BV on PK-15 cells was investigated using a cell viability assay, and up to 150 μM BV did not affect the viability of PK-15 cells at 24 h (Fig. 7A). Subsequently, we analyzed the effect of BV on PCV3 replication. PK-15 cells were pretreated with different concentrations of BV for 1 h, subjected to PCV3 infection, and then cultured for 24 h in PK-15 cells containing BV. The results revealed that BV treatment suppressed PCV3 replication in a dose-dependent manner, as evidenced by a decrease in the expression of Rep protein (Fig. 7B) and virus production (Fig. 7C) compared to levels in the untreated group, suggesting that BV participated in the antiviral activity of HO-1.

BR, a free radical scavenger, plays a key role in antioxidant and anti-inflammatory responses and is rapidly produced by reducing BV through biliverdin reductase (BVR) (46–49). Based on the results of the BV-mediated anti-PCV3 function of HO-1, we explored the effect of BR, a reduction product of BV, on PCV3 replication. Intracellular BR content was first measured in PCV3-infected, or mock-infected PK-15 cells treated or untreated with CoPP or BV for 24 h. The results demonstrated that CoPP or BV significantly increased the BR content, and this activated effect was reversed by PCV3 infection (Fig. 7D and E), indicating that BR may be related to PCV3 replication. To confirm this hypothesis, cell viability was first detected using CCK8 assays in PK-15 cells treated with various concentrations of BR to analyze the cytotoxicity of BR, and the cell viability did not change at concentrations of up to 150 μM (Fig. 7F). Subsequently, PK-15 cells pretreated with various concentrations of BR for 1 h were infected with PCV3 and cultured for 24 h in the presence of BR. As presented in Fig. 7G, the expression of Rep protein decreased in BR-treated PK-15 cells, indicating that BR treatment suppressed PCV3 replication. Similar to the above-described results, viral titers in BR-treated cells exhibited a decreasing trend compared to untreated cells (Fig. 7H). Overall, these results suggest that BR inhibits PCV3 replication.

In most mammalian cells, BV and BVR contribute to the sole metabolic pathway, ultimately resulting in the generation of BR (46, 47), prompting us to analyze if specific siRNA targeting BVR (siBVR) affects the antiviral effect of BV. The Western blotting results revealed that the expression of BVR was silenced by the different siBVR transfections (1, 2, or 3) compared to the control cells (siCon), and siBVR-3 with a notable silencing effect did not exhibit cytotoxicity and was finally selected for use in subsequent experiments (Fig. 7I and J). The effects of BVR knockdown on BR generation from BV were further analyzed. ELISA results demonstrated that the concentration of BR induced by BV was significantly downregulated in PK-15 cells transfected with siBVR (Fig. 7K), indicating that BVR plays a crucial role in BR production from BV. To determine if the antiviral effect of BV was associated with its secondary metabolite BR, PK-15 cells transfected with specific siBVR or siCon were infected with PCV3 and then subjected to treatment with BV, and PCV3 replication was subsequently analyzed. The results demonstrated that silencing of BVR by siBVR increased Rep protein expression and virus production (Fig. 7L and M) compared to the siCon-transfected group, indicating that siBVR transfection partially reversed the inhibitory effect of BV on PCV3 replication.

### The HO-1 upregulation and ROS reduction mediated by BV participate in the anti-PCV3 effect of BV.

**FIG 8:**
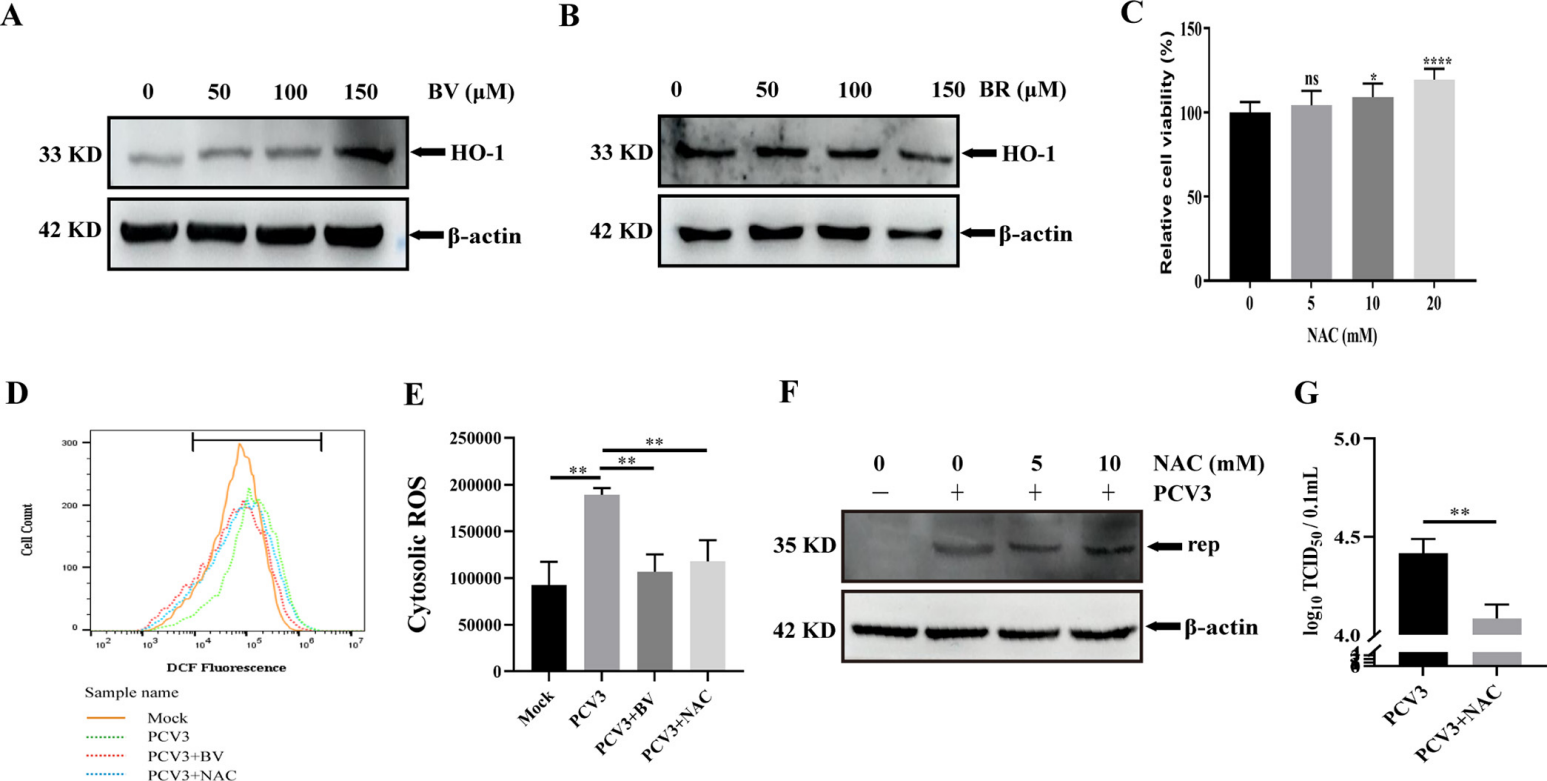
The anti-PCV3 effect of BV depends on the upregulation of HO-1 and the antioxidant activity mediated by BV. (A and B) PK-15 cells were treated or untreated with various concentrations of BV (A) or BR (B) for 24 h and then were collected and measured for HO-1 protein. (C) Cell viability after exposure to different concentrations of NAC (5, 10, and 20 mM) or DMSO (represented by 0 mM NAC) was evaluated using CCK8 assays. (D and E) PK-15 cells were treated or untreated with BV or NAC and then subjected to PCV3 infection, and the cells were assayed for ROS production using the fluorescent probe DCFH-DA. (F and G) PK-15 cells were treated or untreated with NAC and then subjected to PCV3 infection, and the cells and whole-cell culture media were collected and measured for Rep protein (F) and PCV3 titers (G). Data are presented as means ± SD from three independent experiments (ns, *P* > 0.05; *, *P* < 0.05; **, *P* < 0.01; ****, *P* < 0.0001).

To clarify if the antiviral role of BV is associated with HO-1 expression in a positive feedback loop similar to that of CO, the cells were pretreated with different concentrations of BV and then detected by Western blotting. The results revealed that BV treatment enhanced HO-1 expression in a dose-dependent fashion compared to the mock-treated PK-15 cells (Fig. 8A), indicating that the anti-PCV3 effect of BV is related not only to BR but also to the BV-mediated HO-1 increase in a positive feedback regulation. BR, a reduction product of BV (6–9), exhibited the antiviral effect of BV (Fig. 7), prompting us to perform a similar study. However, the results demonstrated that various concentrations of BR did not change the expression level of HO-1 (Fig. 8B), and this was different than to the antiviral effect of BV, indicating that the anti-PCV3 effect of BR did not contribute to HO-1 induction in a positive feedback loop.

It is well established that the protective effects of BV are largely attributed to its antioxidative property (50–52), and viral infection is often accompanied by the generation of reactive oxygen species (ROS) (53–55), resulting in an alteration of the intracellular redox state. Suitable concentrations of *N*-acetylcysteine (NAC), an antioxidative agent and a positive control for ROS decrease, were determined using a cell viability assay. The results showed that the NAC concentration (5 mM) did not affect cell viability (Fig. 8C), and this concentration was then used to evaluate the effect of NAC on ROS production and PCV3 replication. To investigate if BV inhibits PCV3 replication by interfering with ROS production, we first analyzed ROS generation in PCV3-infected PK-15 cells using 2′,7′-dichlorofluorescein diacetate (DCFH-DA), a fluorescent probe, to examine intracellular ROS levels. The results revealed that PCV3 infection increased ROS, whereas BV treatment significantly reduced PCV3-mediated ROS generation (Fig. 8D and E). Further results revealed that NAC attenuated PCV3-mediated ROS production (Fig. 8D and E) and reduced Rep protein expression and viral titers (Fig. 8F and G), and this is different than the inhibitory effect of BV-mediated HO-1 upregulation (Fig. 8A), suggesting that the anti-PCV3 activity of BV also depends on its antioxidative properties.

### The NO-dependent cGMP/PKG signaling pathway participates in the anti-PCV3 effect of BR.

**FIG 9:**
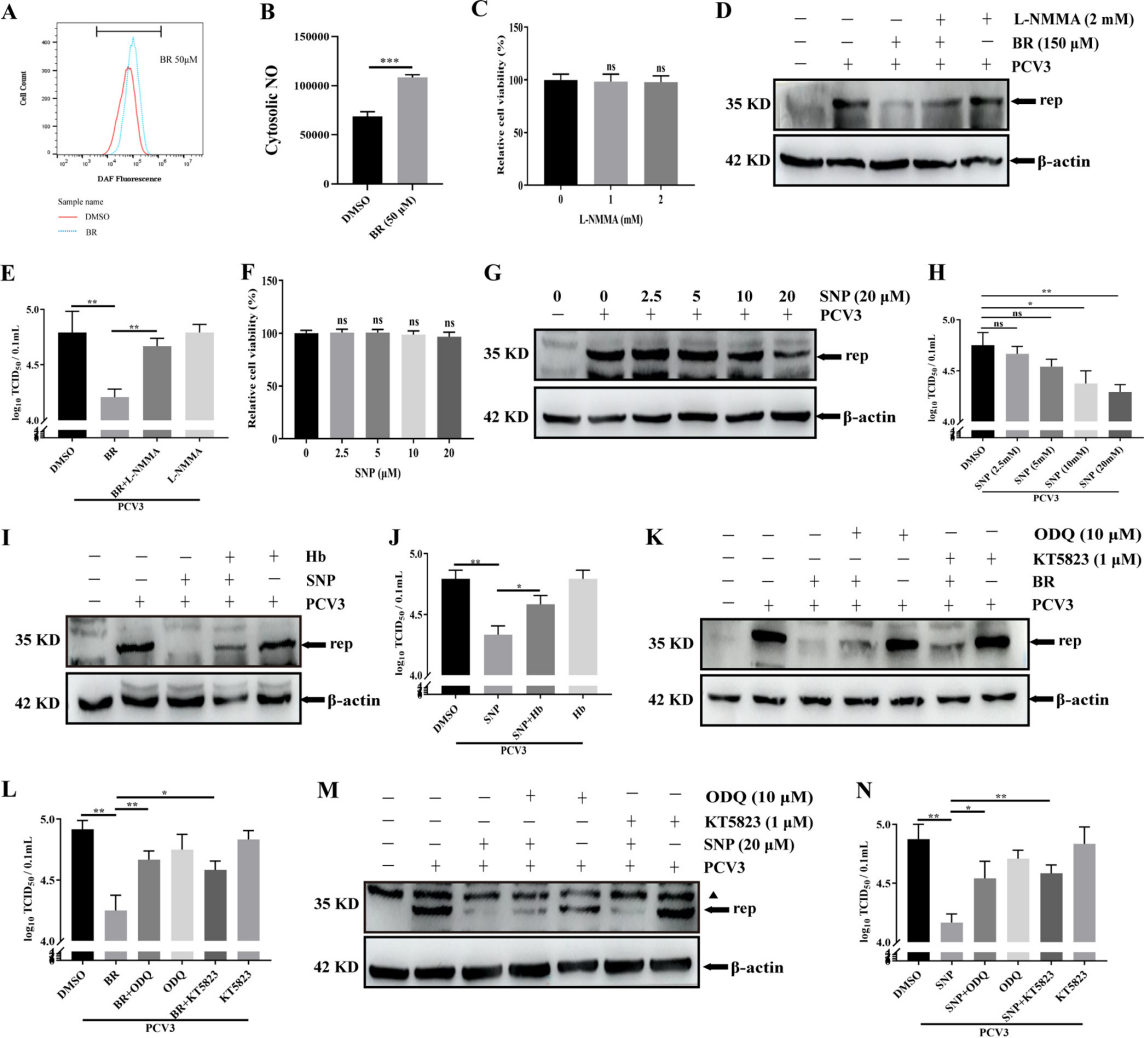
The anti-PCV3 effect of BR depends upon the NO-dependent cGMP/PKG pathway. (A and B) PK-15 cells were treated or untreated with BR for 24 h and then assayed for NO production using the probe DAF-FMDA. (C) Cell viability after exposure to various concentrations of L-NMMA (1 and 2 mM) or DMSO (represented by 0 mM L-NMMA) was evaluated using CCK8 assays. (D and E) PK-15 cells were treated or untreated with L-NMMA or BR and then subjected to PCV3 infection, and the cells and whole-cell culture media were subsequently collected at 24 hpi and measured for Rep protein (D) and PCV3 titers (E). (F) Cell viability after exposure to different concentrations of SNP (2.5, 5, 10, and 20 μM) or DMSO (represented by 0 μM SNP) was evaluated using CCK8 assays. (G and H) PK-15 cells were treated or untreated with SNP and then subjected to PCV3 infection, and the cells and whole-cell culture media were subsequently collected at 24 hpi and measured for Rep protein (G) and PCV3 titers (H). (I and J) PK-15 cells were treated or untreated with SNP or Hb and then subjected to PCV3 infection, and the cells and whole-cell culture media were collected at 24 hpi and measured for Rep protein (I) and PCV3 titers (J). (K and L) PK-15 cells were treated or untreated with BR, ODQ, or KT5823 and then subjected to PCV3 infection, and the cells and whole-cell culture media were collected at 24 hpi and measured for Rep protein (K) and PCV3 titers (L). (M and N) PK-15 cells were treated or untreated with SNP, ODQ, or KT5823 and then subjected to PCV3 infection, and the cells and whole-cell culture media were collected at 24 hpi and measured for Rep protein (M) and PCV3 titers (N); the black triangle represents a nonspecific protein band. Data are expressed as means ± SD from three independent experiments (ns, *P* > 0.05; *, *P* < 0.05; **, *P* < 0.01; ***, *P* < 0.001).

Previous reports have observed that BR promotes NO release by upregulating inducible nitric oxide synthase (iNOS) (56–59). Based on the inhibition of PCV3 replication by BR, we investigated if NO played a crucial role in the antiviral effect of BR. First, NO production was detected in PK-15 cells treated with BR using 3-amino, 4-amino-5-methylamino-2′,7′-di-fluorofluorescein diacetate (DAF-FMDA), a fluorescent probe, to examine intracellular NO levels. As presented in Fig. 9A and B, BR treatment significantly enhanced intracellular NO generation. Subsequently, NG-monomethyl-l-arginine (L-NMMA), a NOS inhibitor, was used to determine a suitable concentration for cell viability and to examine the effect of NO on the inhibition of PCV3 replication by BR. The results demonstrated that cell activity was not influenced by L-NMMA treatment (up to 2 mM) (Fig. 9C), and L-NMMA (2 mM) treatment enhanced Rep protein expression (Fig. 9D) and PCV3 titers (Fig. 9E) compared to those of the L-NMMA-untreated group in PK-15 cells treated with BR, indicating that NO inhibition by L-NMMA partially reversed BR-mediated anti-PCV3 activity in PK-15 cells. Sodium nitroprusside (SNP), an exogenous NO donor, was used to further confirm the anti-PCV3 effect of NO. Nontoxic concentrations of SNP for PK-15 cells were screened using a cell viability assay, and cell activity was not influenced by SNP treatment (up to 20 μM) (Fig. 9F). The Rep protein and PCV3 titers were determined by Western blotting and TCID_50_, respectively. As presented in Fig. 9G and H, SNP treatment decreased PCV3 infection in PK-15 cells dose-dependently. In contrast, Hb, a specific NO scavenger, partially reversed the SNP-mediated inhibitory effect on PCV3-infected PK-15 cells by increasing the expression of the Rep protein (Fig. 9I) and viral titers (Fig. 9J) compared to those of the Hb-untreated group. These data indicate that NO plays a major role in BR-mediated anti-PCV3 activity.

It is well established that sGC is activated and the cGMP content is enhanced in cells producing NO (60), and this prompted us to analyze if the NO-mediated antiviral mechanism is related to sGC activation or the cGMP increase in the cGMP/PKG signaling pathway. This study used specific inhibitors (ODQ and KT5823) of NO-induced sGC or cGMP-dependent protein kinase (PKG) to explore the relationship between sGC, cGMP, and PCV3 replication. As presented in Fig. 9K and L, ODQ or KT5823 markedly blocked the inhibition of PCV3 replication mediated by NO in the presence of BR by enhancing the expression of Rep protein and PCV3 titers. The reversed results of ODQ or KT5823 in the expression of Rep protein and PCV3 titers also were observed in PK-15 cells treated with SNP by blocking NO-mediated sGC or PKG (Fig. 9M and N). These results indicate that the cGMP/PKG signaling pathway contributes to the inhibition of PCV3 infection and plays a crucial role in the NO-mediated anti-PCV3 effect.

### The third metabolite of HO-1 (iron) does not exert an anti-PCV3 function in PK-15 cells.

**FIG 10:**
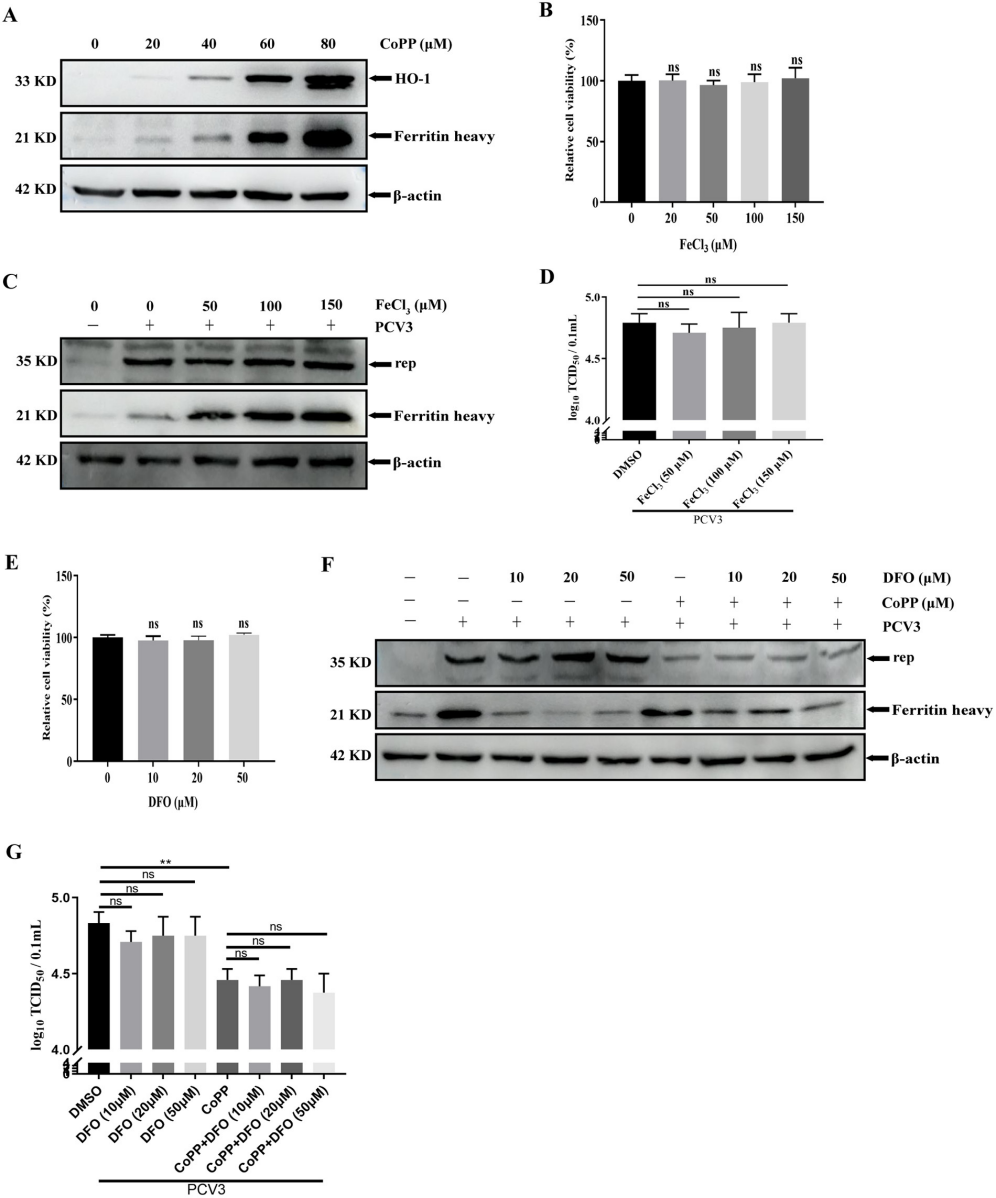
The irons mediated by HO-1 do not affect PCV3 replication. (A) PK-15 cells were treated or untreated with various concentrations of CoPP for 24 h and then collected and measured for HO-1 and ferritin heavy protein. (B) Cell viability after exposure to various concentrations of FeCl_3_ (20, 50, 100, and 150 μM) or water (represented by 0 μM FeCl_3_) was evaluated using CCK8 assays. (C and D) PK-15 cells were treated or untreated with FeCl_3_ and then subjected to PCV3 infection, and the cells and whole-cell culture media were collected at 24 hpi and measured for Rep and ferritin heavy protein (C) and PCV3 titers (D). (E) Cell viability after exposure to various concentrations of DFO (10, 20, and 50 μM) or DMSO (represented by 0 μM DFO) was evaluated using CCK8 assays. (F and G) PK-15 cells were treated or untreated with CoPP or DFO and then subjected to PCV3 infection, and the cells and whole-cell culture media were collected at 24 hpi and measured for Rep and ferritin heavy protein (F) and PCV3 titers (G). Data are presented as means ± SD from three independent experiments (ns, *P* > 0.05; **, *P* < 0.01).

Previous studies have demonstrated that iron is a by-product of heme degradation that mediates the cytoprotective effect of HO-1 (30). CoPP was used as an inducer of HO-1 to analyze the relationship between HO-1 and iron. The results revealed that the various concentrations of CoPP significantly increase HO-1 expression and iron release, as confirmed by the induction of ferritin, an iron storage protein (Fig. 10A) (50). To further explore the effect of iron on PCV3 replication, an iron III chloride solution (FeCl_3_) was selected to mimic the intracellular iron effect. PK-15 cells treated with various concentrations of FeCl_3_ exhibited no differences in cell viability (Fig. 10B) and were used in subsequent experiments to analyze PCV3 replication. As presented in Fig. 10C, FeCl_3_ did not affect the expression of Rep protein while increasing the induction of ferritin (iron storage protein) as an indirect indication of iron, indicating that the increase in intracellular iron did not exert an antiviral effect.

Moreover, the observation of no differences in PCV3 titers between FeCl_3_-treated and untreated PK-15 cells also confirmed the above-described results (Fig. 10D). PK-15 cells treated with various concentrations of deferoxamine (DFO) exhibited no differences in cell viability (Fig. 10E). To determine the effects of endogenous iron on PCV3 replication, DFO, an iron chelator, was used to reduce intracellular iron content in the presence or absence of CoPP, and the level of PCV3 replication was measured. The results revealed that CoPP reduced Rep protein expression and PCV3 titers and that DFO treatment failed to change this reduction in Rep protein and PCV3 titers (Fig. 10F and G), indicating that the decrease in iron chelated by DFO exerted no effect on PCV3 replication. These data indicate that iron, the third metabolite of HO-1, does not exhibit antiviral effects.

## DISCUSSION

**FIG 11:**
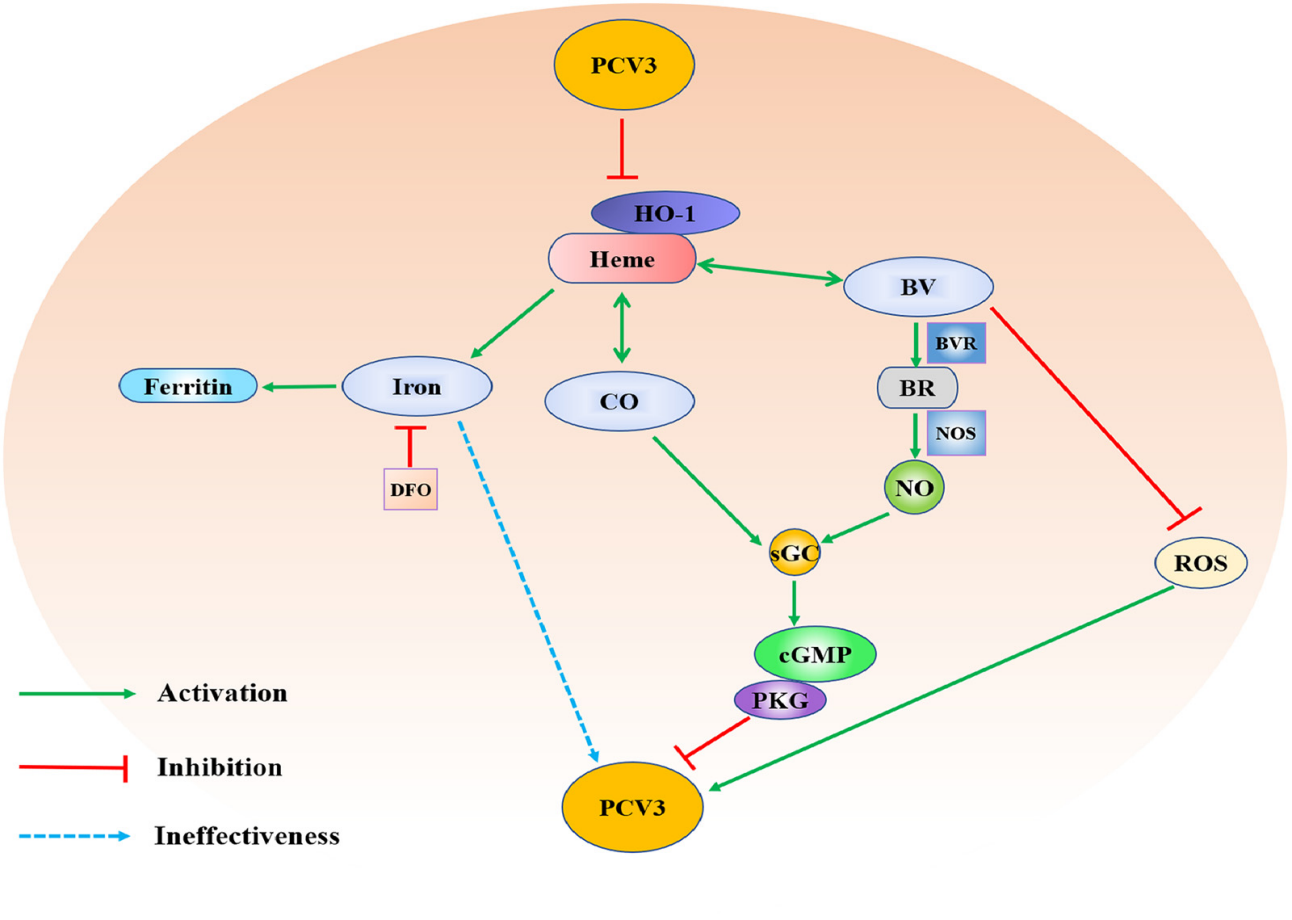
The interaction relationship between PCV3 and HO-1 and its metabolites. PCV3 infection reduces the expression levels of HO-1, and the expression of HO-1 negatively regulates PCV3 replication. The degradation of heme catabolized by HO-1 generates equimolar amounts of CO, BV, and iron (Fe^2+^). CO inhibits PCV3 replication by upregulating HO-1 expression and mediating the cGMP/PKG pathway. BV inhibits PCV3 replication by upregulating HO-1 expression and reducing ROS production and converting it into BR by the enzyme BVR. BR subsequently induces NO production and activates the NO-dependent cGMP/PKG pathway to attenuate PCV3 infection. Both irons released from heme catabolism by HO-1 and iron chelated by DFO do not affect PCV3 replication, and the iron is stored intracellularly by ferritin.

HO-1, an inducible cytoprotective enzyme, is responsible for catalyzing the breakdown of heme that can be regulated in response to various stress stimuli such as oxidative injury, inflammation, and hemorrhage-induced hypoxia and, finally, protects organs or tissues from various types of stresses (61–63). In this protection process, HO-1 induces the production of CO, BV, and iron by degrading heme, utilizing the characteristics of its rate-limiting enzyme, and further exerting its antioxidant and anti-inflammatory properties (64, 65). With increasing research examining HO-1 function, numerous studies have shown the interaction relationship between HO-1 or its enzymatic products (CO, BV, and iron) and viral infection, primarily focusing on the direct inhibition of viral replication. Clarifying the mechanism of HO-1 in the context of PCV3 replication in PK-15 cells is crucial for deepening the understanding of PCV3 infection but also helpful for the development of antiviral drugs. In this study, we determined the anti-PCV3 effect of HO-1 and systematically analyzed the functional pathways of the enzymatic products of HO-1. Our results demonstrated that PCV3 infection downregulated HO-1 expression and that alteration of HO-1 expression negatively regulated PCV3 replication. Moreover, the anti-PCV3 effect of CO or BV primarily depends on the upregulation of HO-1, the reduction of ROS, or the CO- or NO-dependent cGMP/PKG pathway, while iron did not exhibit an inhibitory effect on PCV3 replication (Fig. 11).

Viruses develop various strategies to evade host defense responses, and inhibition of HO-1 expression is an indicator of these strategies. For example, HBV (36), hepatitis C virus (HCV) (1), Zika virus (ZIKV) (66), DENV (40), and PRRSV (50) infection mediated the decrease in HO-1 as a means to facilitate viral infection. Consistent with previous findings, our results revealed that PCV3 infection reduced HO-1 expression in PK-15 cells, and this reduction in HO-1 is associated with active PCV3 replication (Fig. 1), indicating a regulatory relationship between PCV3 replication and HO-1 expression. Subsequently, three criteria were used to further confirm this relationship: (i) CoPP, a specific inducer of HO-1, significantly inhibited PCV3 replication accompanied by an increase in HO-1 expression, and this inhibitory effect of CoPP was specifically modulated by HO-1 (Fig. 2); (ii) HO-1 overexpression using a recombinant lentivirus delivery system inhibited PCV3 replication (Fig. 3A and B); (iii) the knockdown of HO-1 expression by specific siRNA enhanced PCV3 replication (Fig. 3C and D). Our results indicate that the expression of HO-1 negatively regulates PCV3 replication.

The relationship between HO-1 and viral replication varies among viruses. For example, HO-1 suppresses bovine viral diarrhea virus (BVDV), pseudorabies virus (PRV), and PRRSV replication (28, 67, 68) but fails to play an inhibitory role in vesicular stomatitis virus (VSV) infection (39). Furthermore, metabolites of HO-1 and CO have been reported to enhance Kaposi’s sarcoma-associated herpesvirus (KSHV) replication (69). These reports indicate different functions of HO-1 against various viruses. Importantly, these inconsistencies in the effect of HO-1 on viral replication are largely attributed to the unique molecular mechanisms underlying the metabolite of HO-1. The enzymatic activity of HO-1 determines the production of its enzymolysis products (CO, BV, and iron), and these products play a crucial role in regulating cell function, particularly in the context of viral replication (70). ZnPP, an inhibitor of HO-1 enzymatic activity, was exploited to evaluate if HO-1 activity regulated by ZnPP affected PCV3 replication in PK-15 cells. Our results demonstrated that ZnPP treatment partially reversed CoPP-mediated PCV3 inhibition (Fig. 3), indicating that the enzyme activity of HO-1 is essential for PCV3 inhibition.

CO, a metabolite of HO-1 and marker of HO-1 activity, was investigated to determine if it participates in HO-1-mediated PCV3 inhibition and to identify the precise molecular mechanisms. CO is generated by heme degradation by HO-1 (64, 65). HbCO, as a measure of CO (45), was detected in subsequent experiments. Based on our present results, and based on the inhibition of PCV3 replication by CoPP, CoPP treatment improved HbCO content compared to those of the untreated group in PCV3-infected cells (Fig. 5A), and Hb, a CO scavenger (64, 65), partially reversed CoPP-mediated PCV3 attenuation (Fig. 5C and D), suggesting that the antiviral effect by CO is essential for PCV3 infection. Subsequently, CORM-2, a molecule that promotes efficient CO release, was used to further confirm that the increase in CO production inhibited PCV3 replication, whereas iCORM-2 (an inactive variant of CORM-2) did not exhibit a similar inhibition function (Fig. 5G and H). Moreover, treatment with Hb weakened the anti-PCV3 effect of CORM-2 (Fig. 5I and J). These results are consistent with the CO-mediated inhibitory effect on PRRSV and bovine viral diarrhea virus (BVDV) (45, 71), suggesting that CO acts as a downstream metabolite of HO-1 to exert the anti-PCV3 function of HO-1. In recent years, CO, as an important endogenous mediator with signaling properties, has been involved in multiple physiological and pathological processes by regulating critical cellular functions (72). It has been reported that CO induces cGMP production by activating sGC, and this is followed by the regulation of cellular functions such as ion channels, phosphodiesterase, and protein kinases and results in anti-inflammatory effects (73–75). Our results revealed that 8-Br-cGMP, a cGMP analog, inhibited PCV3 replication (Fig. 6B to D), and the inhibition of the cGMP/PKG pathway by ODQ or KT5823 reversed the CORM-2-mediated inhibitory effect on PCV3 replication (Fig. 6E to G), indicating that the CO inhibition of PCV3 infection is dependent on the cGMP/PKG signaling pathway. Similar results were also observed for the inhibitory mechanism of CO on enterovirus 71 (EV71) (43) and PRRSV infection (45). Interestingly, CO suppressed EV71 infection by decreasing cGMP/PKG pathway-dependent NADPH oxidase/reactive oxygen species (ROS) production, while the reduction of ROS induced by NAC inhibited PCV3 replication (Fig. 8F and G), suggesting that the ROS is involved in the inhibition of PCV3 infection. In addition to the above inhibitory mechanisms, our results also demonstrated a positive feedback regulation of CO by CORM-2 treatment on HO-1 expression (Fig. 6A), and this is similar to a previous report (76) but different from PRRSV (45). These data suggest that CO inhibits PCV3 replication by activating the cGMP/PKG signaling pathway and upregulating HO-1 expression. PCV3 infection reduced HO-1 expression, followed by the downregulation of HO-1 activity and CO production, ultimately maintaining its proliferation.

BV, another metabolite of HO-1, participates in a single metabolic pathway to produce BR via BVR (46, 77). Therefore, it is necessary to explore the effect of BV and its reduction product, BR, on PCV3 replication and to clarify the underlying mechanisms. Our results revealed that various concentrations of BV exhibited anti-PCV3 functions (Fig. 7A to C), and BR also exhibited similar effects (Fig. 7F to H), indicating that BV and its reduction product BR generated from HO-1 possess an inhibitory function in PCV3 infection. We next sought to clarify if the anti-PCV3 activity of BV is determined by its reduction product BR. We observed that both CoPP and BV significantly induced BR production (Fig. 7D and E), and knockdown of BVR by specific siRNA targeting BVR reversed BV-mediated inhibition of PCV3 infection (Fig. 7L and M) in a manner that is consistent with PRRSV infection (45), suggesting that BR plays a crucial role in the antiviral effect of BV. The facts that ROS participate in activating and transmitting multiple molecular signaling pathways in cellular and metabolic processes (78, 79) and that BV, a bile pigment, exhibited the capacity to scavenge ROS (45) imply that BV inhibits PCV3 replication by reducing intracellular ROS levels. Our results demonstrated that PCV3 infection enhanced ROS levels in PK-15 cells, and BV treatment downregulated this increase (Fig. 8D and E). Moreover, NAC, a ROS inhibitor, inhibited PCV3 replication by reducing intracellular ROS levels (Fig. 8E to G), and this is similar to our conjecture, indicating that the anti-PCV3 effect of BV is dependent on ROS level. Subsequently, another result demonstrated that BV also possessed a positive feedback regulation on HO-1 expression similar to that of CO, while BR did not (Fig. 8A and B). Based on the importance of BR in the antiviral effects of BV, the inhibitory mechanisms of BR were explored further. BR induces NO generation by neuronal NO synthase (nNOS) in neurons (80), and NO possesses antiviral properties (81, 82), prompting us to explore the relationship between BR and NO and the effect of NO on virus replication in PCV3-infected cells. The experimental results revealed that BR enhanced NO production (Fig. 9A and B), and L-NMMA, a NOS inhibitor, partially weakened the inhibitory effect of NO induced by BR on PCV3 replication (Fig. 9C to E), indicating that BR inhibits PCV3 replication by generating NO. The inhibitory effect of SNP (an NO inducer) on PCV3 replication and Hb (an NO scavenger) partially restored this inhibitory role of SNP, further confirming the above-described conclusion (Fig. 9G to J). NO can activate and interact with guanylate cyclase to induce cGMP generation and initiate intracellular signals (82–84). Our results revealed that the inhibitor of the cGMP/PKG pathway (ODQ or KT5823) weakened BR- or SNP-mediated PCV3 attenuation (Fig. 9K to N), indicating that NO exerts antiviral effects via the cGMP/PKG pathway. Our data suggest that BV inhibits PCV3 replication via HO-1 upregulation, its reduction product BR, and the BR-mediated cGMP/PKG signaling pathway.

In conclusion, this study demonstrated that PCV3 infection downregulates the expression of HO-1 and that HO-1 expression negatively regulates PCV3 replication. The inhibitory mechanisms of HO-1 indicate that its metabolites (CO and BV) mediate the anti-PCV3 effect via HO-1 upregulation, ROS reduction, and the CO- or BR (the reduction product of BV)-dependent cGMP/PKG pathway, whereas iron does not. Clarifying the HO-1-mediated inhibitory effect on PCV3 replication provides important insights for a better understanding of viral pathogenesis and for developing antiviral drugs to prevent and control PCV3 infection.

## MATERIALS AND METHODS

### Cells, viruses, reagents, and antibodies.

PK-15 cells were originally obtained from the American Type Culture Collection (ATCC) and maintained in Dulbecco’s modified Eagle’s medium (DMEM) (Gibco) containing 5% fetal bovine serum (FBS) (Gibco), streptomycin, and penicillin at 37°C in a 5% CO_2_ incubator. The PCV3 LY strain (13) was used in this study.

All reagents and other products were purchased commercially and included cobalt protoporphyrin IX (CoPP, a classic HO-1 inducer) (Santa), zinc-protoporphyrin (ZnPP) (TargetMol), biliverdin (BV) (Topscience), bilirubin (BR) (MedChemExpress), hemoglobin (Hb, a CO or NO scavenger) (Beyotime), tricarbonyldichlororuthenium (II) dimer (CORM-2) (MedChemExpress), 8-Br-cGMP (ApexBio), ODQ (a soluble guanylyl cyclase [sGC] inhibitor) (MedChemExpress), KT5823 (a protein kinase G [PKG] inhibitor) (Beyotime, S1691), sodium nitroferricyanide (III) dihydrate (SNP, a NO donor) (Beyotime), NG-monomethyl-l-arginine (L-NMMA, a NOS inhibitor) (Beyotime), deferoxamine (DFO, an iron chelator) (Sigma), fluorescent probe 2′,7′-dichlorofluorescin diacetate (DCFH-DA) (Sigma-Aldrich), 3-amino, 4-aminomethyl-2′,7′-difluorescein diacetate (DAF-FMDA) (Beyotime), FeCl_3_ (Sbjbio), *N*-acetyl-l-cysteine (NAC) (Beyotime), Cell Counting Kit-8 (CCK8) (Absin), bilirubin (BR) ELISA kit (Elabscience), and porcine carboxyhemoglobin (HbCO) ELISA kit (BMASSAY). Inactive CORM-2 (iCORM-2) is a CORM-2 that liberates CO by incubating CORM-2 dissolved in dimethyl sulfoxide (DMSO) for 24 h at 37°C in a 5% CO_2_ incubator.

Pig anti-PCV3 Rep antibody was obtained from our laboratory, and other antibodies were purchased commercially, including rabbit anti-heme oxygenase 1 (HO-1) antibody (Abcam) and rabbit anti-ferritin antibody (ABclonal). Rabbit anti-BVR antibody (ABclonal), mouse anti-β-actin antibody (Sangon Biotech), and rabbit anti-GFP antibody (Huabio) were also used.

### Lentivirus transduction and the construction of cell lines expressing green fluorescent protein (GFP)-HO-1 or GFP.

The HO-1 gene from PK-15 cells was amplified and cloned into the lentivirus vector pWPXL to express GFP-tagged HO-1 (GFP-HO-1). Three lentivirus packaging plasmids, pWPXL-GFP-HO-1 or pWPXL, pMD2.G, and psPAX2, were cotransfected into HEK293T cells using Lipofectamine 2000 (Invitrogen) following the manufacturer’s protocol, and the supernatants were collected at 48 h posttransfection, filtered, and concentrated. PK-15 cells in 24-well plates were transduced with concentrated lentiviruses for 36 h, and GFP-expressing cells were sorted into single wells of a 96-well plate. Several single-cell clones were cultured for 3 weeks, identified, and used in subsequent experiments.

### Viral infection and TCID_50_ assay.

PK-15 cells cultured to a confluence of approximately 90% were pretreated with various concentrations of reagents (CoPP, ZnPP, Hb, CORM-2, iCORM-2, 8-Br-cGMP, ODQ, KT5823, BV, BR, NAC, L-NMMA, SNP, FeCl_3_, and DFO) at 37°C and then infected with PCV3 at a multiplicity of infection (MOI) of 0.5 for 1 h at 37°C. The cells treated with different reagents were processed for different experiments. The whole-cell culture medium was collected at the indicated times in PCV3-infected cells and was assayed by immunofluorescence assay using an immunofluorescence microscope (Olympus IX73, Florida, USA) for viral titers by serial dilution using Spearman and Karber’s method and was represented as the 50% tissue culture infectious dose (TCID_50_) as in the case of PCV2 (85, 86).

### RNA extraction and reverse transcriptase quantitative PCR (RT-qPCR).

Total RNAs were extracted from PK-15 cells using TRIzol reagent (Invitrogen) following the manufacturer’s protocol, and cDNA synthesis was conducted using the Vazyme cDNA synthesis kit with the corresponding primers. cDNA was detected thrice using the *Taq* Pro universal SYBR quantitative qPCR master mix kit (Vazyme) (Roche LightCycler real-time PCR detection system). The 2^−ΔΔ^*^CT^* method was used to evaluate the relative expression of the mRNAs of different genes. The primer sequences (5′–3′) for HO-1 and glyceraldehyde-3-phosphate dehydrogenase (GAPDH) in RT-qPCR are as follows: qHO-1-F (5′-TACCGCTCCCGAATGAACAC-3′), qHO-1-R (5′-CCTTAGTGTCCTGGGTCAGC-3′), qGAPDH-F (5′-TCGGAGTGAACGGATTTGGC-3′), and qGAPDH-R (5′-TGACAAGCTTCCCGTTCTCC-3′).

### Western blotting.

Proteins were extracted from PK-15 cells using radioimmunoprecipitation assay (RIPA) lysis buffer (Beyotime) and quantified using a bicinchoninic acid protein assay kit (Thermo). Then, 20 μg of total proteins was separated by sodium dodecyl sulfate-polyacrylamide gel electrophoresis (SDS-PAGE) and transferred to nitrocellulose membranes (Pall). The membranes were blocked in phosphate-buffered saline (PBS) with 5% nonfat milk for 2 h at room temperature, incubated with primary antibody or horseradish peroxidase (HRP)-conjugated secondary antibody for 2 h, and then exposed using a Super Signal West Pico PLUS chemiluminescent substrate kit (Thermo) in a chemiluminescence apparatus (Amersham).

### Cell viability assay.

The effects of various inhibitors on cell viability were determined using CCK8. Briefly, PK-15 cells were pretreated with various concentrations of reagents and processed with the corresponding reagents, and cell viability was then measured at the indicated times following the manufacturer’s protocols.

### ELISA detection for intracellular HbCO or BR.

PK-15 cells were grown on 6-well plates, treated with the indicated concentrations of CoPP or BV, and/or infected with PCV3. After incubation for 24 h, cells were collected, lysed using NP40 lysis buffer (Beyotime), and centrifuged (12,000 × *g*, 20 min, 4°C). The supernatants were measured using an HbCO or BR ELISA kit in the dark following the manufacturer’s protocol. Absorbance was measured at 450 nm using an Infinite M Plex instrument (Tecan).

### siRNA transfection.

siRNAs targeting the HO-1 and BVR genes were designed or purchased from GenePharma and transfected using Lipofectamine RNAiMAX (Thermo) according to the manufacturer’s protocol for 36 to 48 h. PK-15 cells were infected with PCV3, followed by treatment with CoPP or BV. Cell samples, whole-cell culture medium, or cell supernatants were harvested at the indicated times and analyzed using Western blotting, TCID_50_, or ELISA. siHO-1 (sense, 5′-GGACAUGGCCUUCUGGUAUTT-3′; antisense, 5′-AUACCAGAAGGCCAUGUCCTT-3′), siBVR-1 (sense, 5′-GGAGCAUUGAGGAGGUGAATT-3′; antisense, 5′-UUCACCUCCUCAAUGCUCCTT-3′), siBVR-2 (sense, 5′-GCCUCAAGCGAAACAGACATT-3′; antisense, 5′-UGUCUGUUUCGCUUGAGGCTT-3′), and siBVR-3 (sense, 5′-GCCGCCGAGAAGAAACGUATT-3′; antisense, 5′-UACGUUUCUUCUCGGCGGCTT-3′).

### Measurement of ROS or NO levels.

Cytosolic ROS or NO in PCV3-infected PK-15 cells treated with NAC (pH 7.4), BV, or BR were harvested after trypsin treatment, collected, resuspended in Hanks’ balanced salt solution (HBSS) (Beyotime), and probed with 10 μM 2′,7′-dichlorodihydrofluorescein diacetate (DCFH-DA) or 5 μM 3-amino, 4-aminomethyl-2′,7′-di-fluorescein diacetate (DAF-FMDA) for 30 min at 37°C in the dark. The cells were washed with HBSS and analyzed by flow cytometry using a CytoFLEX flow cytometer (Beckmann).

### Statistical analysis.

Statistical differences for all data were determined by one-way analysis of variance (ANOVA) or Student’s *t* test using Prism 9.0 software (GraphPad Software, San Diego, CA, USA), with a *P* value of <0.05 being considered statistically significant.

## References

[1] Abdalla MY, Britigan BE, Wen F, Icardi M, McCormick ML, LaBrecque DR, Voigt M, Brown KE, Schmidt WN. 2004. Down-regulation of heme oxygenase-1 by hepatitis C virus infection in vivo and by the in vitro expression of hepatitis C core protein. J Infect Dis 190:1109–1118. doi:10.1086/423488. 15319861

[2] Palinski R, Pineyro P, Shang P, Yuan F, Guo R, Fang Y, Byers E, Hause BM. 2017. A novel porcine circovirus distantly related to known circoviruses is associated with porcine dermatitis and nephropathy syndrome and reproductive failure. J Virol 91:e01879-16. doi:10.1128/JVI.01879-16. 27795441 PMC5165205

[3] Wen S, Sun W, Li Z, Zhuang X, Zhao G, Xie C, Zheng M, Jing J, Xiao P, Wang M, Han J, Ren J, Liu H, Lu H, Jin N. 2018. The detection of porcine circovirus 3 in Guangxi, China. Transbound Emerg Dis 65:27–31. doi:10.1111/tbed.12754. 29119691

[4] Tochetto C, Lima DA, Varela APM, Loiko MR, Paim WP, Scheffer CM, Herpich JI, Cerva C, Schmitd C, Cibulski SP, Santos AC, Mayer FQ, Roehe PM. 2018. Full-genome sequence of porcine circovirus type 3 recovered from serum of sows with stillbirths in Brazil. Transbound Emerg Dis 65:5–9. doi:10.1111/tbed.12735. 29027372

[5] Kedkovid R, Woonwong Y, Arunorat J, Sirisereewan C, Sangpratum N, Lumyai M, Kesdangsakonwut S, Teankum K, Jittimanee S, Thanawongnuwech R. 2018. Porcine circovirus type 3 (PCV3) infection in grower pigs from a Thai farm suffering from porcine respiratory disease complex (PRDC). Vet Microbiol 215:71–76. doi:10.1016/j.vetmic.2018.01.004. 29426409

[6] Stadejek T, Woźniak A, Miłek D, Biernacka K. 2017. First detection of porcine circovirus type 3 on commercial pig farms in Poland. Transbound Emerg Dis 64:1350–1353. doi:10.1111/tbed.12672. 28649803

[7] Kwon T, Yoo SJ, Park CK, Lyoo YS. 2017. Prevalence of novel porcine circovirus 3 in Korean pig populations. Vet Microbiol 207:178–180. doi:10.1016/j.vetmic.2017.06.013. 28757021

[8] Faccini S, Barbieri I, Gilioli A, Sala G, Gibelli LR, Moreno A, Sacchi C, Rosignoli C, Franzini G, Nigrelli A. 2017. Detection and genetic characterization of porcine circovirus type 3 in Italy. Transbound Emerg Dis 64:1661–1664. doi:10.1111/tbed.12714. 28921870

[9] Fux R, Sockler C, Link EK, Renken C, Krejci R, Sutter G, Ritzmann M, Eddicks M. 2018. Full genome characterization of porcine circovirus type 3 isolates reveals the existence of two distinct groups of virus strains. Virol J 15:25. doi:10.1186/s12985-018-0929-3. 29378597 PMC5789634

[10] Franzo G, Legnardi M, Hjulsager CK, Klaumann F, Larsen LE, Segales J, Drigo M. 2018. Full-genome sequencing of porcine circovirus 3 field strains from Denmark, Italy and Spain demonstrates a high within-Europe genetic heterogeneity. Transbound Emerg Dis 65:602–606. doi:10.1111/tbed.12836. 29453822

[11] Li G, He W, Zhu H, Bi Y, Wang R, Xing G, Zhang C, Zhou J, Yuen KY, Gao GF, Su S. 2018. Origin, genetic diversity, and evolutionary dynamics of novel porcine circovirus 3. Adv Sci (Weinh) 5:1800275. doi:10.1002/advs.201800275. 30250786 PMC6145280

[12] Fu X, Fang B, Ma J, Liu Y, Bu D, Zhou P, Wang H, Jia K, Zhang G. 2018. Insights into the epidemic characteristics and evolutionary history of the novel porcine circovirus type 3 in southern China. Transbound Emerg Dis 65:e296–e303. doi:10.1111/tbed.12752. 29178283

[13] Jiang H, Wang D, Wang J, Zhu S, She R, Ren X, Tian J, Quan R, Hou L, Li Z, Chu J, Guo Y, Xi Y, Song H, Yuan F, Wei L, Liu J. 2019. Induction of porcine dermatitis and nephropathy syndrome in piglets by infection with porcine circovirus type 3. J Virol 93:e02045-18. doi:10.1128/JVI.02045-18. 30487279 PMC6363995

[14] Hou L, Wang J, Zhang W, Quan R, Wang D, Zhu S, Jiang H, Wei L, Liu J. 2020. Dynamic alterations of gut microbiota in porcine circovirus type 3-infected piglets. Front Microbiol 11:1360. doi:10.3389/fmicb.2020.01360. 32714299 PMC7341976

[15] Sirisereewan C, Thanawongnuwech R, Kedkovid R. 2022. Current understanding of the pathogenesis of porcine circovirus 3. Pathogens 11:64. doi:10.3390/pathogens11010064. 35056012 PMC8778431

[16] Shi R, Hou L, Wei L, Quan R, Zhou B, Jiang H, Wang J, Zhu S, Song J, Wang D, Liu J. 2021. Porcine circovirus type 3 enters into PK15 cells through clathrin- and dynamin-2-mediated endocytosis in a Rab5/Rab7 and pH-dependent fashion. Front Microbiol 12:636307. doi:10.3389/fmicb.2021.636307. 33679671 PMC7928314

[17] Song J, Hou L, Wang D, Wei L, Zhu S, Wang J, Quan R, Jiang H, Shi R, Liu J. 2021. Nucleolar phosphoprotein NPM1 interacts with porcine circovirus type 3 Cap protein and facilitates viral replication. Front Microbiol 12:679341. doi:10.3389/fmicb.2021.679341. 34113334 PMC8185148

[18] Shen H, Liu X, Zhang P, Wang S, Liu Y, Zhang L, Song C. 2020. Porcine circovirus 3 Cap inhibits type I interferon signaling through interaction with STAT2. Virus Res 275:197804. doi:10.1016/j.virusres.2019.197804. 31697988

[19] Liu X, Shen H, Zhang X, Liang T, Ban Y, Yu L, Zhang L, Liu Y, Dong J, Zhang P, Lian K, Song C. 2021. Porcine circovirus type 3 capsid protein induces NF-kappaB activation and upregulates pro-inflammatory cytokine expression in HEK-293T cells. Arch Virol 166:2141–2149. doi:10.1007/s00705-021-05104-z. 34009439

[20] Jiang H, Wei L, Wang D, Wang J, Zhu S, She R, Liu T, Tian J, Quan R, Hou L, Li Z, Chu J, Zhou J, Guo Y, Xi Y, Song H, Yuan F, Liu J. 2020. ITRAQ-based quantitative proteomics reveals the first proteome profiles of piglets infected with porcine circovirus type 3. J Proteomics 212:103598. doi:10.1016/j.jprot.2019.103598. 31785380

[21] Ewing JF, Maines MD. 1995. Distribution of constitutive (HO-2) and heat-inducible (HO-1) heme oxygenase isozymes in rat testes: HO-2 displays stage-specific expression in germ cells. Endocrinology 136:2294–2302. doi:10.1210/endo.136.5.7720678. 7720678

[22] Hsu M, Muchova L, Morioka I, Wong RJ, Schroder H, Stevenson DK. 2006. Tissue-specific effects of statins on the expression of heme oxygenase-1 in vivo. Biochem Biophys Res Commun 343:738–744. doi:10.1016/j.bbrc.2006.03.036. 16563347

[23] Choi AM, Alam J. 1996. Heme oxygenase-1: function, regulation, and implication of a novel stress-inducible protein in oxidant-induced lung injury. Am J Respir Cell Mol Biol 15:9–19. doi:10.1165/ajrcmb.15.1.8679227. 8679227

[24] Ewing JF, Haber SN, Maines MD. 1992. Normal and heat-induced patterns of expression of heme oxygenase-1 (HSP32) in rat brain: hyperthermia causes rapid induction of mRNA and protein. J Neurochem 58:1140–1149. doi:10.1111/j.1471-4159.1992.tb09373.x. 1737989

[25] Wu L, Wang R. 2005. Carbon monoxide: endogenous production, physiological functions, and pharmacological applications. Pharmacol Rev 57:585–630. doi:10.1124/pr.57.4.3. 16382109

[26] Hayashi S, Omata Y, Sakamoto H, Higashimoto Y, Hara T, Sagara Y, Noguchi M. 2004. Characterization of rat heme oxygenase-3 gene. Implication of processed pseudogenes derived from heme oxygenase-2 gene. Gene 336:241–250. doi:10.1016/j.gene.2004.04.002. 15246535

[27] Waza AA, Hamid Z, Ali S, Bhat SA, Bhat MA. 2018. A review on heme oxygenase-1 induction: is it a necessary evil. Inflamm Res 67:579–588. doi:10.1007/s00011-018-1151-x. 29693710

[28] Zhang A, Wan B, Jiang D, Wu Y, Ji P, Du Y, Zhang G. 2020. The cytoprotective enzyme heme oxygenase-1 suppresses pseudorabies virus replication in vitro. Front Microbiol 11:412. doi:10.3389/fmicb.2020.00412. 32231654 PMC7082841

[29] Zhu Z, Wilson AT, Mathahs MM, Wen F, Brown KE, Luxon BA, Schmidt WN. 2008. Heme oxygenase-1 suppresses hepatitis C virus replication and increases resistance of hepatocytes to oxidant injury. Hepatology 48:1430–1439. doi:10.1002/hep.22491. 18972446 PMC2587102

[30] Ryter SW, Alam J, Choi AM. 2006. Heme oxygenase-1/carbon monoxide: from basic science to therapeutic applications. Physiol Rev 86:583–650. doi:10.1152/physrev.00011.2005. 16601269

[31] Otterbein LE, Foresti R, Motterlini R. 2016. Heme oxygenase-1 and carbon monoxide in the heart: the balancing act between danger signaling and pro-survival. Circ Res 118:1940–1959. doi:10.1161/CIRCRESAHA.116.306588. 27283533 PMC4905590

[32] Thomas DT, DelCimmuto NR, Flack KD, Stec DE, Hinds TD Jr. 2022. Reactive oxygen species (ROS) and antioxidants as immunomodulators in exercise: implications for heme oxygenase and bilirubin. Antioxidants (Basel) 11:179. doi:10.3390/antiox11020179. 35204062 PMC8868548

[33] McClung JA, Levy L, Garcia V, Stec DE, Peterson SJ, Abraham NG. 2022. Heme-oxygenase and lipid mediators in obesity and associated cardiometabolic diseases: Therapeutic implications. Pharmacol Ther 231:107975. doi:10.1016/j.pharmthera.2021.107975. 34499923 PMC8958338

[34] Reizenstein P. 1991. Iron, free radicals and cancer. Med Oncol Tumor Pharmacother 8:229–233. doi:10.1007/BF02987191. 1820488

[35] Chung SW, Hall SR, Perrella MA. 2009. Role of haem oxygenase-1 in microbial host defence. Cell Microbiol 11:199–207. doi:10.1111/j.1462-5822.2008.01261.x. 19016784 PMC3080039

[36] Protzer U, Seyfried S, Quasdorff M, Sass G, Svorcova M, Webb D, Bohne F, Hosel M, Schirmacher P, Tiegs G. 2007. Antiviral activity and hepatoprotection by heme oxygenase-1 in hepatitis B virus infection. Gastroenterology 133:1156–1165. doi:10.1053/j.gastro.2007.07.021. 17919491

[37] Espinoza JA, Leon MA, Cespedes PF, Gomez RS, Canedo-Marroquin G, Riquelme SA, Salazar-Echegarai FJ, Blancou P, Simon T, Anegon I, Lay MK, Gonzalez PA, Riedel CA, Bueno SM, Kalergis AM. 2017. Heme oxygenase-1 modulates human respiratory syncytial virus replication and lung pathogenesis during infection. J Immunol 199:212–223. doi:10.4049/jimmunol.1601414. 28566367

[38] Devadas K, Dhawan S. 2006. Hemin activation ameliorates HIV-1 infection via heme oxygenase-1 induction. J Immunol 176:4252–4257. doi:10.4049/jimmunol.176.7.4252. 16547262

[39] Xiao S, Zhang A, Zhang C, Ni H, Gao J, Wang C, Zhao Q, Wang X, Wang X, Ma C, Liu H, Li N, Mu Y, Sun Y, Zhang G, Hiscox JA, Hsu WH, Zhou EM. 2014. Heme oxygenase-1 acts as an antiviral factor for porcine reproductive and respiratory syndrome virus infection and over-expression inhibits virus replication in vitro. Antiviral Res 110:60–69. doi:10.1016/j.antiviral.2014.07.011. 25086213

[40] Tseng CK, Lin CK, Wu YH, Chen YH, Chen WC, Young KC, Lee JC. 2016. Human heme oxygenase 1 is a potential host cell factor against dengue virus replication. Sci Rep 6:32176. doi:10.1038/srep32176. 27553177 PMC4995454

[41] Kikuchi G, Yoshida T, Noguchi M. 2005. Heme oxygenase and heme degradation. Biochem Biophys Res Commun 338:558–567. doi:10.1016/j.bbrc.2005.08.020. 16115609

[42] Blancou P, Tardif V, Simon T, Remy S, Carreno L, Kalergis A, Anegon I. 2011. Immunoregulatory properties of heme oxygenase-1. Methods Mol Biol 677:247–268. doi:10.1007/978-1-60761-869-0_18. 20941616

[43] Tung WH, Hsieh HL, Lee IT, Yang CM. 2011. Enterovirus 71 induces integrin beta1/EGFR-Rac1-dependent oxidative stress in SK-N-SH cells: role of HO-1/CO in viral replication. J Cell Physiol 226:3316–3329. doi:10.1002/jcp.22677. 21321939

[44] Li Y, Gao C, Shi Y, Tang Y, Liu L, Xiong T, Du M, Xing M, Liu L, Yao P. 2013. Carbon monoxide alleviates ethanol-induced oxidative damage and inflammatory stress through activating p38 MAPK pathway. Toxicol Appl Pharmacol 273:53–58. doi:10.1016/j.taap.2013.08.019. 23994557

[45] Zhang A, Zhao L, Li N, Duan H, Liu H, Pu F, Zhang G, Zhou EM, Xiao S. 2017. Carbon monoxide inhibits porcine reproductive and respiratory syndrome virus replication by the cyclic GMP/protein kinase G and NF-kappaB signaling pathway. J Virol 91:e01866-16. doi:10.1128/JVI.01866-16. 27795439 PMC5165190

[46] Maines MD. 2005. New insights into biliverdin reductase functions: linking heme metabolism to cell signaling. Physiology (Bethesda) 20:382–389. doi:10.1152/physiol.00029.2005. 16287987

[47] Kapitulnik J, Maines MD. 2009. Pleiotropic functions of biliverdin reductase: cellular signaling and generation of cytoprotective and cytotoxic bilirubin. Trends Pharmacol Sci 30:129–137. doi:10.1016/j.tips.2008.12.003. 19217170

[48] Lerner-Marmarosh N, Miralem T, Gibbs PE, Maines MD. 2008. Human biliverdin reductase is an ERK activator; hBVR is an ERK nuclear transporter and is required for MAPK signaling. Proc Natl Acad Sci USA 105:6870–6875. doi:10.1073/pnas.0800750105. 18463290 PMC2383961

[49] Lerner-Marmarosh N, Shen J, Torno MD, Kravets A, Hu Z, Maines MD. 2005. Human biliverdin reductase: a member of the insulin receptor substrate family with serine/threonine/tyrosine kinase activity. Proc Natl Acad Sci USA 102:7109–7114. doi:10.1073/pnas.0502173102. 15870194 PMC1088173

[50] Zhang A, Duan H, Li N, Zhao L, Pu F, Huang B, Wu C, Nan Y, Du T, Mu Y, Zhao Q, Sun Y, Zhang G, Hiscox JA, Zhou EM, Xiao S. 2017. Heme oxygenase-1 metabolite biliverdin, not iron, inhibits porcine reproductive and respiratory syndrome virus replication. Free Radic Biol Med 102:149–161. doi:10.1016/j.freeradbiomed.2016.11.044. 27908781

[51] Foresti R, Goatly H, Green CJ, Motterlini R. 2001. Role of heme oxygenase-1 in hypoxia-reoxygenation: requirement of substrate heme to promote cardioprotection. Am J Physiol Heart Circ Physiol 281:H1976–H1984. doi:10.1152/ajpheart.2001.281.5.H1976. 11668058

[52] Clark JE, Foresti R, Sarathchandra P, Kaur H, Green CJ, Motterlini R. 2000. Heme oxygenase-1-derived bilirubin ameliorates postischemic myocardial dysfunction. Am J Physiol Heart Circ Physiol 278:H643–H651. doi:10.1152/ajpheart.2000.278.2.H643. 10666097

[53] Yan Y, Xin A, Liu Q, Huang H, Shao Z, Zang Y, Chen L, Sun Y, Gao H. 2015. Induction of ROS generation and NF-kappaB activation in MARC-145 cells by a novel porcine reproductive and respiratory syndrome virus in Southwest of China isolate. BMC Vet Res 11:232. doi:10.1186/s12917-015-0480-z. 26358082 PMC4565009

[54] Ferrari M, Zevini A, Palermo E, Muscolini M, Alexandridi M, Etna MP, Coccia EM, Fernandez-Sesma A, Coyne C, Zhang DD, Marques ETA, Olagnier D, Hiscott J. 2020. Dengue virus targets Nrf2 for NS2B3-mediated degradation leading to enhanced oxidative stress and viral replication. J Virol 94:e01551-20. doi:10.1128/JVI.01551-20. 32999020 PMC7925186

[55] Xia N, Wang H, Liu X, Shao Q, Ao D, Xu Y, Jiang S, Luo J, Zhang J, Chen N, Meurens F, Zheng W, Zhu J. 2020. African swine fever virus structural protein p17 inhibits cell proliferation through ER stress-ROS mediated cell cycle arrest. Viruses 13:21. doi:10.3390/v13010021. 33374251 PMC7823474

[56] Mancuso C, Bonsignore A, Di Stasio E, Mordente A, Motterlini R. 2003. Bilirubin and S-nitrosothiols interaction: evidence for a possible role of bilirubin as a scavenger of nitric oxide. Biochem Pharmacol 66:2355–2363. doi:10.1016/j.bcp.2003.08.022. 14637193

[57] Mancuso C, Bonsignore A, Capone C, Di Stasio E, Pani G. 2006. Albumin-bound bilirubin interacts with nitric oxide by a redox mechanism. Antioxid Redox Signal 8:487–494. doi:10.1089/ars.2006.8.487. 16677092

[58] Genc S, Genc K, Kumral A, Baskin H, Ozkan H. 2003. Bilirubin is cytotoxic to rat oligodendrocytes in vitro. Brain Res 985:135–141. doi:10.1016/s0006-8993(03)03037-3. 12967717

[59] Sarady-Andrews JK, Liu F, Gallo D, Nakao A, Overhaus M, Ollinger R, Choi AM, Otterbein LE. 2005. Biliverdin administration protects against endotoxin-induced acute lung injury in rats. Am J Physiol Lung Cell Mol Physiol 289:L1131–L1137. doi:10.1152/ajplung.00458.2004. 16155084

[60] Schulz S, Chinkers M, Garbers DL. 1989. The guanylate cyclase/receptor family of proteins. FASEB J 3:2026–2035. doi:10.1096/fasebj.3.9.2568301. 2568301

[61] Applegate LA, Luscher P, Tyrrell RM. 1991. Induction of heme oxygenase: a general response to oxidant stress in cultured mammalian cells. Cancer Res 51:974–978. 1988141

[62] Hashiba T, Suzuki M, Nagashima Y, Suzuki S, Inoue S, Tsuburai T, Matsuse T, Ishigatubo Y. 2001. Adenovirus-mediated transfer of heme oxygenase-1 cDNA attenuates severe lung injury induced by the influenza virus in mice. Gene Ther 8:1499–1507. doi:10.1038/sj.gt.3301540. 11593363

[63] Vallabhaneni R, Kaczorowski DJ, Yaakovian MD, Rao J, Zuckerbraun BS. 2010. Heme oxygenase 1 protects against hepatic hypoxia and injury from hemorrhage via regulation of cellular respiration. Shock 33:274–281. doi:10.1097/SHK.0b013e3181b0f566. 19536046

[64] McCoubrey WK Jr, Huang TJ, Maines MD. 1997. Isolation and characterization of a cDNA from the rat brain that encodes hemoprotein heme oxygenase-3. Eur J Biochem 247:725–732. doi:10.1111/j.1432-1033.1997.00725.x. 9266719

[65] Ryter SW, Choi AM. 2005. Heme oxygenase-1: redox regulation of a stress protein in lung and cell culture models. Antioxid Redox Signal 7:80–91. doi:10.1089/ars.2005.7.80. 15650398

[66] El Kalamouni C, Frumence E, Bos S, Turpin J, Nativel B, Harrabi W, Wilkinson DA, Meilhac O, Gadea G, Despres P, Krejbich-Trotot P, Viranaicken W. 2018. Subversion of the heme oxygenase-1 antiviral activity by Zika virus. Viruses 11:2. doi:10.3390/v11010002. 30577437 PMC6356520

[67] Zhang C, Pu F, Zhang A, Xu L, Li N, Yan Y, Gao J, Liu H, Zhang G, Goodfellow IG, Zhou EM, Xiao S. 2015. Heme oxygenase-1 suppresses bovine viral diarrhoea virus replication in vitro. Sci Rep 5:15575. doi:10.1038/srep15575. 26510767 PMC4625146

[68] Hill-Batorski L, Halfmann P, Neumann G, Kawaoka Y. 2013. The cytoprotective enzyme heme oxygenase-1 suppresses Ebola virus replication. J Virol 87:13795–13802. doi:10.1128/JVI.02422-13. 24109237 PMC3838215

[69] Botto S, Gustin JK, Moses AV. 2017. The heme metabolite carbon monoxide facilitates KSHV infection by inhibiting TLR4 signaling in endothelial cells. Front Microbiol 8:568. doi:10.3389/fmicb.2017.00568. 28421060 PMC5376558

[70] Ma LL, Sun L, Wang YX, Sun BH, Li YF, Jin YL. 2021. Association between HO1 gene promoter polymorphisms and diseases (Review). Mol Med Rep 25:29. doi:10.3892/mmr.2021.12545. 34841438 PMC8669660

[71] Ma Z, Pu F, Zhang X, Yan Y, Zhao L, Zhang A, Li N, Zhou EM, Xiao S. 2017. Carbon monoxide and biliverdin suppress bovine viral diarrhoea virus replication. J Gen Virol 98:2982–2992. doi:10.1099/jgv.0.000955. 29087274

[72] Ghosh S, Gal J, Marczin N. 2010. Carbon monoxide: endogenous mediator, potential diagnostic and therapeutic target. Ann Med 42:1–12. doi:10.3109/07853890903482877. 20092397

[73] Morita T, Perrella MA, Lee ME, Kourembanas S. 1995. Smooth muscle cell-derived carbon monoxide is a regulator of vascular cGMP. Proc Natl Acad Sci USA 92:1475–1479. doi:10.1073/pnas.92.5.1475. 7878003 PMC42542

[74] Lucas KA, Pitari GM, Kazerounian S, Ruiz-Stewart I, Park J, Schulz S, Chepenik KP, Waldman SA. 2000. Guanylyl cyclases and signaling by cyclic GMP. Pharmacol Rev 52:375–414. 10977868

[75] Jackson EB Jr, Mukhopadhyay S, Tulis DA. 2007. Pharmacologic modulators of soluble guanylate cyclase/cyclic guanosine monophosphate in the vascular system: from bench top to bedside. Curr Vasc Pharmacol 5:1–14. doi:10.2174/157016107779317224. 17266609 PMC11795701

[76] Yang CM, Lin CC, Lee IT, Hsu CK, Tai YC, Hsieh HL, Chi PL, Hsiao LD. 2015. c-Src-dependent transactivation of EGFR mediates CORM-2-induced HO-1 expression in human tracheal smooth muscle cells. J Cell Physiol 230:2351–2361. doi:10.1002/jcp.24912. 25921464

[77] Maines MD. 1997. The heme oxygenase system: a regulator of second messenger gases. Annu Rev Pharmacol Toxicol 37:517–554. doi:10.1146/annurev.pharmtox.37.1.517. 9131263

[78] Bachschmid M, Schildknecht S, Ullrich V. 2005. Redox regulation of vascular prostanoid synthesis by the nitric oxide-superoxide system. Biochem Biophys Res Commun 338:536–542. doi:10.1016/j.bbrc.2005.08.157. 16153593

[79] Ullrich V, Kissner R. 2006. Redox signaling: bioinorganic chemistry at its best. J Inorg Biochem 100:2079–2086. doi:10.1016/j.jinorgbio.2006.09.019. 17095095

[80] Mancuso C, Capone C, Ranieri SC, Fusco S, Calabrese V, Eboli ML, Preziosi P, Galeotti T, Pani G. 2008. Bilirubin as an endogenous modulator of neurotrophin redox signaling. J Neurosci Res 86:2235–2249. doi:10.1002/jnr.21665. 18338802

[81] Akaike T, Maeda H. 2000. Nitric oxide and virus infection. Immunology 101:300–308. doi:10.1046/j.1365-2567.2000.00142.x. 11106932 PMC2327086

[82] Croen KD. 1993. Evidence for antiviral effect of nitric oxide. Inhibition of herpes simplex virus type 1 replication. J Clin Invest 91:2446–2452. doi:10.1172/JCI116479. 8390481 PMC443304

[83] Ignarro LJ. 1991. Signal transduction mechanisms involving nitric oxide. Biochem Pharmacol 41:485–490. doi:10.1016/0006-2952(91)90618-f. 1847633

[84] Moncada S, Palmer RM, Higgs EA. 1991. Nitric oxide: physiology, pathophysiology, and pharmacology. Pharmacol Rev 43:109–142. 1852778

[85] Fenaux M, Halbur PG, Haqshenas G, Royer R, Thomas P, Nawagitgul P, Gill M, Toth TE, Meng XJ. 2002. Cloned genomic DNA of type 2 porcine circovirus is infectious when injected directly into the liver and lymph nodes of pigs: characterization of clinical disease, virus distribution, and pathologic lesions. J Virol 76:541–551. doi:10.1128/jvi.76.2.541-551.2002. 11752145 PMC136831

[86] Wei L, Kwang J, Wang J, Shi L, Yang B, Li Y, Liu J. 2008. Porcine circovirus type 2 induces the activation of nuclear factor kappa B by IkappaBalpha degradation. Virology 378:177–184. doi:10.1016/j.virol.2008.05.013. 18561971

