# Supplementary material for: Heme Oxygenase-1 and Its Metabolites Carbon Monoxide and Biliverdin, but Not Iron, Exert Antiviral Activity against Porcine Circovirus Type 3
Source: Microbiol Spectr. 2023 May 4;11(3):e05060-22. doi: 10.1128/spectrum.05060-22 (PMC10269822; doi:10.1128/spectrum.05060-22)
Supplement: Supplemental file 1 — Fig. S1. Download spectrum.05060-22-s0001.pdf, PDF file, 0.2 MB [file spectrum.05060-22-s0001.pdf]

Fig. S1. PK-15 cell lines stably overexpressing GFP-HO-1 or GFP were constructed using a recombinant lentiviral delivery system. (A) PK-15 cell lines stably overexpressing GFP-HO-1 or GFP were screened and cultured. (B) PK-15 cell lines stably overexpressing GFP-HO-1 or GFP were collected and analyzed for GFP-HO-1 or GFP protein by western blotting. (C) Viability of PK-15 cell lines stably overexpressing GFP-HO-1 or GFP was evaluated using the CCK8 assay. Data are presented as mean ± SD from three independent experiments (ns, $P > 0.05$).

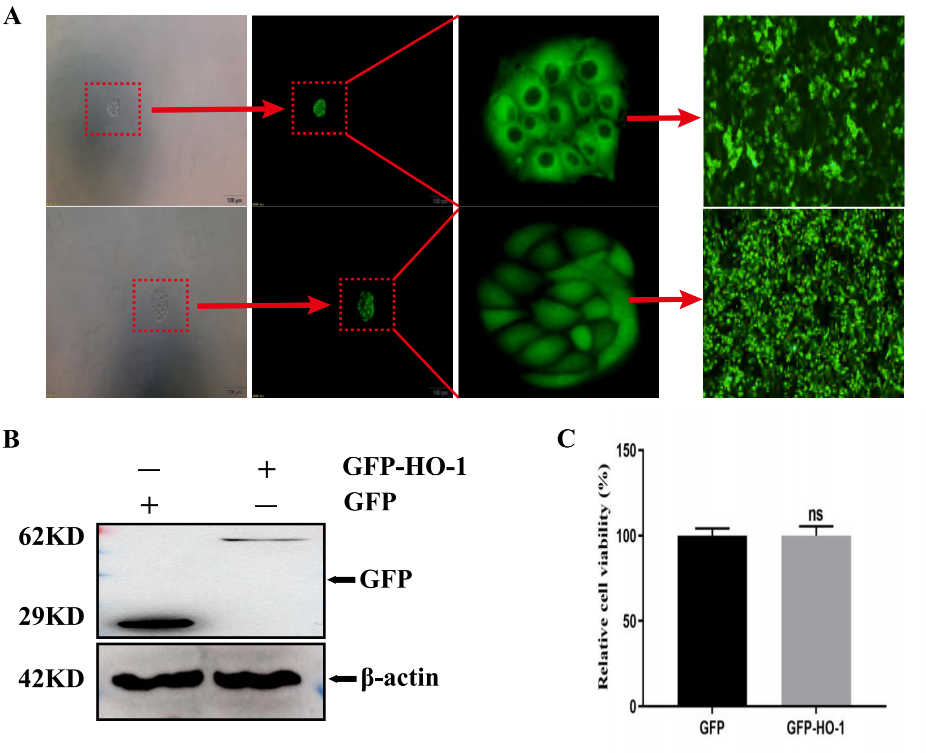
A
B
—
+
GFP-HO-1
+
—
GFP
62KD
29KD
42KD
GFP
β-actin
C
Relative cell viability (%)
150
100
50
0
ns
GFP
GFP-HO-1
